# The *O*-Glycome of Human Nigrostriatal Tissue and Its Alteration in Parkinson’s Disease

**DOI:** 10.1021/acs.jproteome.1c00219

**Published:** 2021-06-30

**Authors:** Hayden Wilkinson, Kristina A. Thomsson, Ana L. Rebelo, Mark Hilliard, Abhay Pandit, Pauline M. Rudd, Niclas G. Karlsson, Radka Saldova

**Affiliations:** † NIBRT GlycoScience Group, National Institute for Bioprocessing, Research and Training, Blackrock, Dublin A94 X099, Ireland; ‡ CÚRAM, SFI Research Centre for Medical Devices, National University of Ireland, Galway, Galway H91 W2TY, Ireland; § UCD School of Medicine, College of Health and Agricultural Science, University College Dublin, Dublin D07 A8NN, Ireland; ∥ Department of Medical Biochemistry and Cell Biology, Institute of Biomedicine, Sahlgrenska Academy, University of Gothenburg, Gothenburg 405 30, Sweden; ⊥ Department of Life Sciences and Health, Faculty of Health Sciences, Oslo Metropolitan University, Oslo 0167, Norway

**Keywords:** methods, *O*-glycans, β-elimination, liquid chromatography (LC), mass spectrometry (MS), exoglycosidases, striatum, substantia nigra, Parkinson’s disease (PD), incidental Lewy bodies disease (ILBD), biomarkers

## Abstract

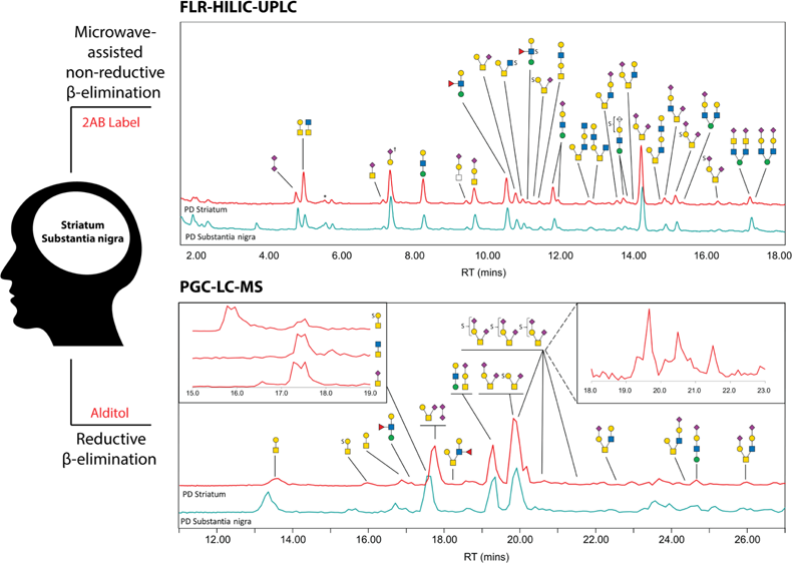

*O*-Glycosylation changes in misfolded proteins are of particular interest in understanding neurodegenerative conditions such as Parkinson’s disease (PD) and incidental Lewy body disease (ILBD). This work outlines optimizations of a microwave-assisted *nonreductive* release to limit glycan degradation and employs this methodology to analyze *O*-glycosylation on the human striatum and substantia nigra tissue in PD, ILBD, and healthy controls, working alongside well-established *reductive* release approaches. A total of 70 *O*-glycans were identified, with ILBD presenting significantly decreased levels of mannose-core (*p* = 0.017) and glucuronylated structures (*p* = 0.039) in the striatum and PD presenting an increase in sialylation (*p* < 0.001) and a decrease in sulfation (*p* = 0.001). Significant increases in sialylation (*p* = 0.038) in PD were also observed in the substantia nigra. This is the first study to profile the whole nigrostriatal *O*-glycome in healthy, PD, and ILBD tissues, outlining disease biomarkers alongside benefits of employing orthogonal techniques for *O*-glycan analysis.

## Introduction

Glycosylation is the most common post-translational modification of macromolecules in eukaryotes, influencing cell–cell interactions, protein folding and stability, and in receptor binding and recognition.^1^ The most common classes of protein glycosylation are *N*-glycans and *O*-glycans.^2^ The common pentasaccharide core of *N*-glycans (GlcNAc_2_Man_3_) is recognized by several endoglycosidases,^3^ allowing cleavage, enrichment/separation, and analysis. *O*-Glycans present much more of a challenge due to the absence of a suitable deglycosylating enzyme that targets the range of *O*-glycan cores. Alkaline release reagents are instead used to release *O*-glycans *via* two common approaches, reductive and nonreductive β-elimination.^4^ The former uses a strong base and reducing agent to generate glycan alditols, and the latter incorporates a milder base in the presence of an “end-cap” reagent to generate an intermediate species susceptible to fluorescence derivatization. A downside is an alkaline-driven “peeling” reaction, which degrades the glycan.^5^ Microwave methods have been proposed to limit this by reducing reaction times.^6^

Glycans analyzed by fluorescence are often referenced against a dextran homopolymer standard to contrive their corresponding glucose units (GUs) which are compared against fluorescence-based databases,^7^ and exoglycosidase digestions using linkage-specific enzymes allow determination of glycan composition and linkages.^8^ Electrospray ionization (ESI)^9^ and matrix-assisted laser desorption/ionization^10^ MS linked with tandem mass spectrometry (MS/MS) approaches such as collision-induced dissociation (CID), higher energy CID, and reactive electron excitation (ExD) are used for in-depth glycan structural determination.

Parkinson’s disease (PD) is a neurodegenerative disorder symptomized by bradykinesia, dementia, and depression^11^ and is linked to the degeneration of dopaminergic neurons in the *substantia nigra pars compacta* due in large part to inflammation, oxidative stress, and mitochondrial and the ubiquitin-proteasome pathway dysfunction.^12−14^ These factors relate to the aggregation and misfolding of proteins, which play a cardinal role under the most neurodegenerative conditions. Of interest is the role that the presynaptic neuronal protein α-synuclein plays in PD neuropathy. During PD degeneration, Lewy neurites and globular Lewy bodies form through toxic α-synuclein aggregation causing neuroinflammation, degeneration, and lesions and ultimately cause cell death.^15,16^ As glycosylation is known to affect protein folding and the structure, glycans may be important in the PD pathogenesis and identified as potential biomarkers. Braak *et al.* proposed a staging concept for PD diagnosis based on synuclein pathology and abnormal synuclein manifestation and has since become widely accepted.^17^ Six stages separate the degree of parkinsonism, stages 1 and 2 are limited to lesions in the dorsal motor nucleus of the medulla oblongata and locus coeruleus and are defined as incidental Lewy body disease (ILBD).^18^ However, ILBD may be rather associated with aging as Lewy bodies are seen in ∼10% of clinically normal people.^19^ In stages 3 and 4 PD, toxic α-synuclein pathology reaches the substantia nigra amygdala and the mesocortex, and in stages 5 and 6, the prefrontal and primary neocortices are affected.^20^

Lower sialylation and higher fucosylation in triantennary glycans have been identified in PD serum *N*-glycans,^21^ and decreases in oligomannose structures and an increase in core-fucosylated monogalactosylated structures have been seen in PD IgG *N*-glycans.^22^*O*-GlcNAcylation on α-synuclein proteins associated with PD was found to be increased, corresponding to a decrease in toxic α-synuclein aggregation.^23^ Up to 30% of *O*-glycans from associated tissue contain a core mannose residue, derived from glycoproteins including α-dystroglycan, which extend often by the addition of *N*-acetylglucosamine, galactose, fucose, sialic acid, and the uncommon 3-*O*-sulfated glucuronic acid residue, generating potential 50–60 structures.^24,25^ The glycosylation of α-dystroglycan is known to play a vital role in the correct muscle function, with hypoglycosylation causing congenital muscular dystrophy (CMD) and being associated with cancer metastasis.^26^ Core 1 mucin-type structures were found to be differentially regulated in the PD mice model, in midbrain, stratum, and hippocampus regions with an accumulation of core 1 glycans on microtubule-associated protein 6 in the disease state,^27^ hypothesized to damage neurons within the dopaminergic pathway.

The whole *O*-glycomes in PD- and ILBD-affected tissue in humans have yet to be analyzed in depth. Herein, we present optimizations to a novel microwave-assisted ammonia-based nonreductive *O*-glycan release permitting fluorescent labeling and analysis with limited peeling by increasing release speed through exposure to microwave radiation (minimizing the time spent in a basic degradation-prone solution). This optimized method was then employed for subsequent *O*-glycan analysis on FLR-HILIC-UPLC and FLR-HILIC-UPLC-MS, coupled with reductive β-elimination approaches and analysis using PGC-LC-ESI-MS^*n*^, as well as exoglycosidase digestions. Significant *O*-glycosylation changes occurring both spatially and temporally after the onset of disease from the whole tissue from nigrostriatal brain regions in control, PD, and ILBD postmortem patients were identified.

## Materials and Methods

### Initial Sample Preparation

H_2_O in the following experiments is from a Milli-Q (Millipore) system with a resistivity of 18.2 MΩ·cm (at 25 °C). For optimization and reproducibility studies of the nonreductive release method, fetuin glycoprotein (Sigma-Aldrich) and porcine gastric mucin (Sigma-Aldrich) were used. For positive controls in PD/ILBD study, fetuin glycoprotein was used. For reproducibility studies, as well as definitive studies on human tissue, the frozen autopsied striatum and substantia nigra from 33 patients with PD or ILBD, healthy matched controls, and associated clinical and neuropathological data were provided by the Parkinson’s UK Brain Bank, funded by Parkinson’s UK, a charity registered in England and Wales (258197) and in Scotland (SC037554). The age, gender, and Braak stages of the different samples are described in Table S1. The frozen brain tissue (approximately 30 mg per tissue containing approximately 1 mg of the total protein) was homogenized in RIPA buffer and cOmplete protease inhibitor cocktail (Roche) through mechanical disruption using Qiagen TissueLyser LT, at a frequency of 40 Hz, 4 °C, for an 8 min duration. The homogenates were centrifuged for 20 min at 13,000 rpm, 4 °C, and the supernatants were collected and kept at −80 °C until further analysis. Expected protein content for glycan analysis was roughly 1 mg per tissue sample.

### *N*-Glycan Release

The glycoprotein standard and final tissue samples were prepared for release using a serum release method described by Royle *et al.*(28) modified for in-tube workup of whole tissue samples using an in-gel procedure described by Samal *et al.*,^29^ and *N*-glycans were released using PNGase F as previously described.^30^ The remaining gels after *N*-glycan elution were separated (75% for nonreductive and 25% for reductive *O*-glycan release).

### *O*-Glycan Release—Microwave-Assisted Nonreductive β-Elimination

Gels from *N*-glycan release were crushed by passing them through a small hole in the bottom of a 0.2 mL tube into a 1.5 mL tube (under 14,000 rpm centrifugal force), dried down, resuspended in 250 μL 40% dimethylamine in water containing 0.1 g/mL ammonium carbonate, and transferred to G4 vials (Anton Paar). Tubes were subjected to 12 min microwave radiation at 70 °C in a Monowave 450 microwave reactor at 600 W (Anton Paar). The reaction solution, including gel, was transferred to 2 mL tubes and neutralized with 1 M HCl. Samples were desalted using a HyperSep Hypercarb SPE cartridge and dried.

### Glycan Labeling with 2AB

Nonreductively released *O*-glycans were fluorescently labeled through reductive amination with 2AB.^31^ The excess label was removed using a HyperSep Diol SPE cartridge.^32^

### *O*-Glycan Release—Reductive β-Elimination

*O*-Glycans were released using β-elimination and desalted according to Schulz *et al.*(33) (modified for an in-gel release).^34^

### FLR-WAX-UPLC Analysis of 2AB-Labeled Glycans

2AB-labeled *O*-glycans were resuspended in 50 μL of water, and 20 μL was injected into an Acquity H-class ultraperformance liquid chromatography system (Waters Corporation) coupled with a fluorescence detector (Waters Corporation). The oligosaccharides were separated on a DEAE anion-exchange 75 × 7.5 mm i.d., 10 μm particle size column (Waters Corporation) at a flow rate of 750 μL/min and a column at ambient temperature. The oligosaccharides were eluted using buffer A [20% v/v acetonitrile (ACN)] and buffer B (0.1 M ammonium acetate pH 7.0; Sigma-Aldrich, in 20% ACN) over a 30 min run using the following gradient: 0.00–5.00 min—100% A, 5.00–20.00 min—100% → 0% A, 20.00–22.50 min—0% A, 22.50–23.00 min—0% → 100% A, and 23.00–30.00 min—100% A. Fluorescence was measured at 420 nm, with excitation at 330 nm. External referencing was performed by comparing fluorescence trace against 2AB-labeled fetuin *O*-glycans (Ludger).

### FLR-HILIC-UPLC Analysis of 2AB-Labeled Glycans

Nonreductive β-eliminated 2AB-labeled *O*-glycans were resuspended in 20 μL of 88% ACN, and 19 μL was injected into an Acquity H-class hydrophilic-interaction liquid chromatography-ultraperformance liquid chromatography (HILIC-UPLC) system (Waters Corporation) coupled with a fluorescence detector (Waters Corporation). The oligosaccharides were separated on an ACQUITY UPLC glycan BEH amide, 130 Å, 1.7 μm column (Waters Corporation) at a flow rate of 561 μL/min and a column temperature of 40 °C. The oligosaccharides were eluted using buffer A (50 mM ammonium formate, prepared with 50 mM formic acid, adjusted to pH 4.4 with ammonium hydroxide solution) and buffer B (ACN) over a 30 min run using the following gradient: 0.00–1.47 min—12% A, 1.47–25.00 min—12% → 47.6% A, 25.00–25.60 min—47.6% → 70% A (flow rate 300 μL/min), 25.60–26.80 min—70% A (flow rate 300 μL/min), 26.80–28.00 min—70% → 12% A (flow rate 300 μL/min), and 28.00–30.00 min—12% A (flow rate 561 μL/min). Fluorescence was measured at 420 nm, with excitation at 330 nm. External calibration was performed using 2AB-labeled glucose oligomers, creating a dextran ladder with retention times (RTs) of all identified glycan peaks expressed in GUs.

### FLR-HILIC-UPLC-MS Analysis of 2AB-Labeled Glycans

2AB-labeled *O*-glycans were desalted using 10 μL of normal-phase PhyTip columns (PhyNexus Inc.). *O*-Glycans were resuspended in 10 μL of 88% ACN, and 9 μL was injected into an Acquity H-class HILIC-UPLC system (Waters Corporation) with a BEH glycan column (1.0 × 150 mm, 1.7 μm particle size; Waters Corporation) and an Acquity fluorescence detector coupled inline with a Waters Xevo G2 QTof system (Waters Corporation). The flow rate was 150 μL/min, and the column temperature was maintained at 60 °C. The oligosaccharides were eluted using buffer A (50 mM ammonium formate, prepared with 50 mM formic acid, adjusted to pH 4.4 with ammonium hydroxide solution) and buffer B (ACN) over a 30 min run using the following gradient: 0.00–1.00 min—12% A, 1.00–25.00 min—12% → 47% A, 25.00–25.50 min—47% → 70% A, 25.50–25.55 min—70% A (flow rate 100 μL/min), 25.55–26.50 min—70% A (flow rate 100 μL/min), 26.50–27.00 min—70% → 12% A (flow rate 100 μL/min), and 27.00–30.00 min—12% A (flow rate 150 μL/min). Fluorescence was measured at 420 nm, with excitation at 330 nm. For MS acquisition data, the instrument was operated in the negative-ion sensitivity mode with a capillary voltage of 1.80 kV. The ion source block and nitrogen desolvation gas temperatures were set at 120 and 400 °C, respectively. The desolvation gas was set to a flow rate of 600 L/h. The cone voltage was maintained at 50 V. Full-scan data for glycans were acquired over the *m*/*z* range of 450 to 2500. Data collection and processing were controlled using MassLynx 4.1 software (Waters Corporation).

### PGC-LC-MS^*n*^ Analysis of Glycan Alditols

Reductive β-eliminated *O*-glycan alditols were resuspended in 20 μL of water, and 2 μL was injected onto a liquid chromatography-ESI tandem mass spectrometry (LC-ESI-MS) system with a 10 cm × 250 μm column packed in-house with 5 μm porous graphite particles (Thermo-Hypersil). The flow rate was 5 μL/min, and the column was at ambient temperature. The oligosaccharides were eluted using buffer A [10 mM ammonium bicarbonate (ABC)] and buffer B (10 mM ABC in 80% ACN) using the following gradient: 0.00–46.00 min—100% → 55% A, 46.00–54.00 min—55% → 0% A, and 54.00–78.00 min—0% → 100% A. The samples were analyzed in the negative-ion mode on an LTQ linear ion trap mass spectrometer (Thermo Electron), with an IonMax standard ESI source equipped with a stainless-steel needle kept at −3.5 kV. Compressed air was used as the nebulizer gas. The heated capillary was kept at 270 °C, and the capillary voltage was −50 kV. Full scan (*m*/*z* 380–1800, two microscans, maximum 100 ms, and a target value of 30,000) was performed, followed by data-dependent MS^2^ or MS^3^ scans (two microscans, maximum 100 ms, and a target value of 10,000) with a normalized collision energy of 30%, isolation window of 2.5 units, activation *q* = 0.25, and activation time 30 ms. The thresholds for MS^2^ and MS^3^ were set to 300 and 100 counts, respectively. Data acquisition was conducted with Xcalibur 2.0.7 software (Thermo Scientific).

### Exoglycosidase Arrays

2AB-labeled *O*-glycans were subjected to digestion using an exoglycosidase panel as described by Saldova *et al.* 2014^35^ using arrays of the following enzymes: α2-3 sialidase cloned from *Streptococcus pneumoniae* and expressed in *Escherichia coli* (NAN1, EC 3.2.1.18), 5 U/mL; α2-3,6,8,9 sialidase cloned from *Arthrobacter ureafaciens* and expressed in *E. coli* (ABS, EC 3.2.1.18), 1000 U/mL; β1-3,4 galactosidase cloned from bovine testis and expressed in *Pichia pastoris* (BTG, EC 3.2.1.23), 200 U/mL; β1-4 galactosidase cloned from *S. pneumoniae* and expressed in *E. coli* (SPG, EC 3.2.1.23), 80 U/mL; α1-2,3,4,6 fucosidase cloned from bovine kidney and expressed in *E. coli* (BKF, EC 3.2.1.51), 800 U/mL; β1-2,3,4,6 *N*-acetylglucosaminidase cloned from *S. pneumoniae* and expressed in *E. coli* (GUH, EC 3.2.1.30), 400 U/mL; α1-2,3,6 mannosidase cloned from *Canavalia ensiformis* (jack bean) and expressed in *P. pastoris* (JBM, EC 3.2.1.24), 400 U/mL; α1-3,4 fucosidase cloned from the sweet almond tree (*Prunus dulcis*) and expressed in *P. pastoris* (AMF, EC 3.2.1.111), 400 mU/mL; β1-3,4,6-*N*-acetylhexosaminidase (β1-4 for GalNAc only) cloned from *Streptomyces plicatus* and overexpressed in *E. coli* (JBH, EC 3.2.1.52), 800 U/mL; and α1-3,4,6 galactosidase cloned from green coffee bean and expressed in *E. coli* (CBG, EC 3.2.1.22), 800 U/mL. All enzymes were from New England Biolabs (Hitchin, Herts, U.K.) except for NAN1 which was purchased from Prozyme (San Leandro, CA).

### Statistical Analysis

Glycan peak area data for individuals within each group were logit-transformed, as described by Saldova *et al.* 2014.^35^ Peak data were checked for normal distribution using a Kolmogorov–Smirnov nonparametric test. Multivariate analysis of variance (MANOVA) with *post hoc* analysis using Tukey’s honest significant difference test was then performed using SPSS (IBM) to identify significant peak differences across groups (*p* < 0.05 was considered statistically significant). Principal component analysis (PCA) was performed on % peak area and % feature area of each sample/group using the Perseus software platform^36^ to identify relatedness of patient groups and samples. Spatial and temporal differences were compared upon the onset of PD, as well as differences in gender (M/F) and age (below 85, 85+).

## Results

### Development of a Microwave-Assisted Nonreductive β-Elimination *O*-Glycan Release

Multiple parameters were tested across the *O*-glycan release protocol, as outlined in the Materials and Methods and Table S2. The coefficient of variation (CV) for the major FLR-HILIC-UPLC chromatogram peaks in fetuin and porcine gastric mucin replicates was below 20% using the optimized method (Figure S1). CVs for 10 major chromatogram peaks in replicates from a PD substantia nigra pool were below 15% (Table S3).

### Whole *O*-Glycome Characterization in Human PD and ILBD Tissues

**Figure 1:**
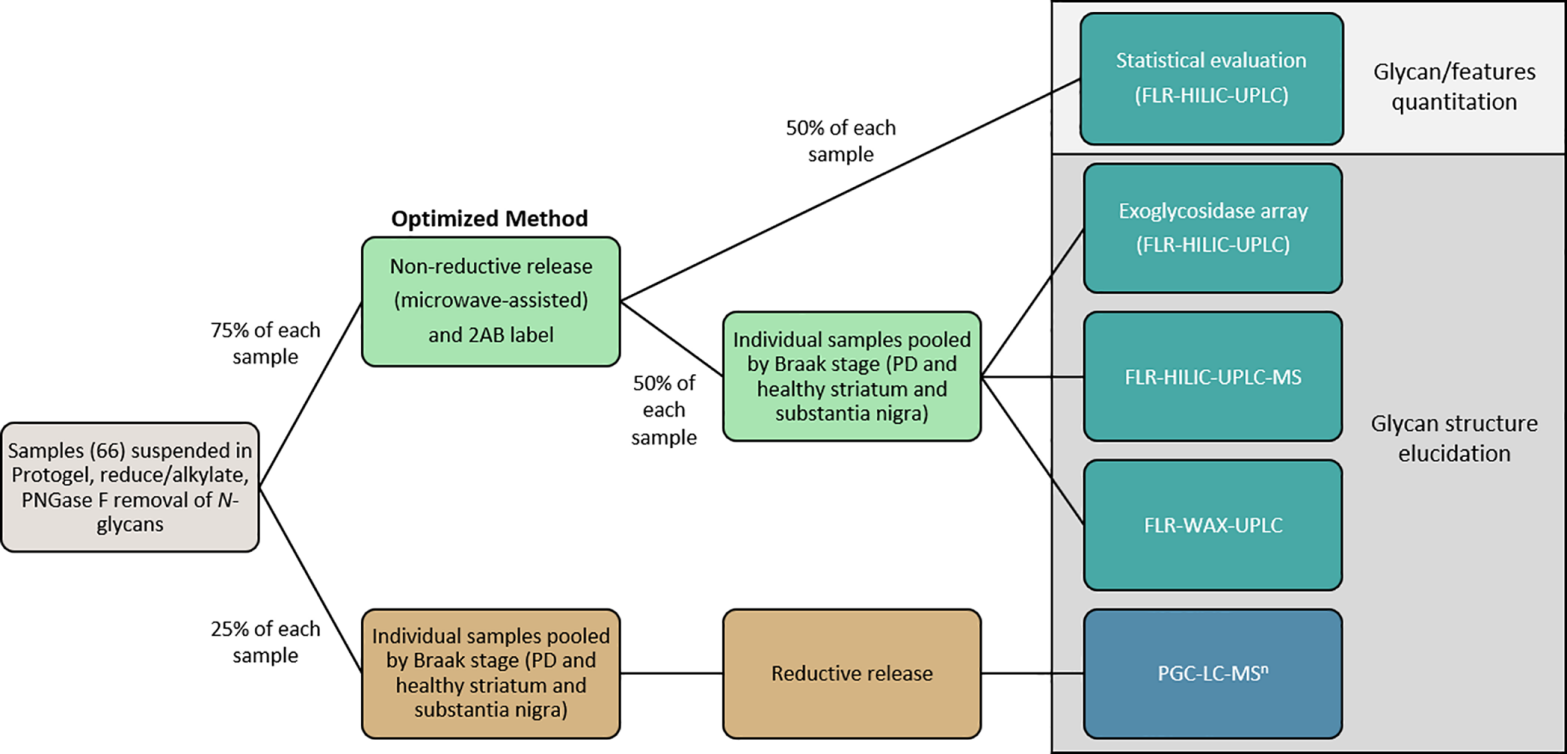
Workflow schematic of sample preparation and analysis incorporating both reductive (lower brown track) and nonreductive (upper green track) β-elimination techniques and analytical approaches. In total, 66 samples were set in the gel and had *N*-glycans cleaved and separated. 25% of each sample gel was pooled into four respective groups—healthy striatum, PD striatum (containing Braak stages 1–4), healthy substantia nigra, and PD substantia nigra (containing Braak stages 1–4). The combination of ILBD (stages 1–2) and PD (stages 3–4) was required due to low sample numbers from ILBD (three patients—three striata and three substantia nigra samples). Healthy groups acted as the control for each brain region. These pools were subjected to reductive β-elimination, and recovered glycan alditols were identified using MS^1^, MS^2^, and MS^3^ analyses to confirm composition and linkage position. With knowledge of reduced glycan profiles for each pool, the remaining 75% of each sample gel was subjected to microwave-assisted nonreductive β-elimination and 2AB labeling. 50% of each sample glycan release was analyzed by FLR-HILIC-UPLC for statistical evaluation. The remaining 50% of released glycans were pooled into their respective groups and subjected to exoglycosidase digestions (followed by another round of FLR-HILIC-UPLC analysis) and orthogonal FLR-HILIC-UPLC-MS^1^ analysis to corroborate exoglycosidase findings, referenced against glycan alditols analyzed by PGC-LC-MS^*n*^. FLR-WAX-UPLC analysis was also performed to confirm glycan charge distribution across the profile for each group.

Individual samples were prepared for the glycan analysis using the developed optimized method for FLR-HILIC-UPLC/FLR-WAX-UPLC and exoglycosidase digestions in combination with the established reductive β-elimination,^33^ linked to PGC-LC-MS^*n*^ according to the scheme in Figure 1.

**Figure 2:**
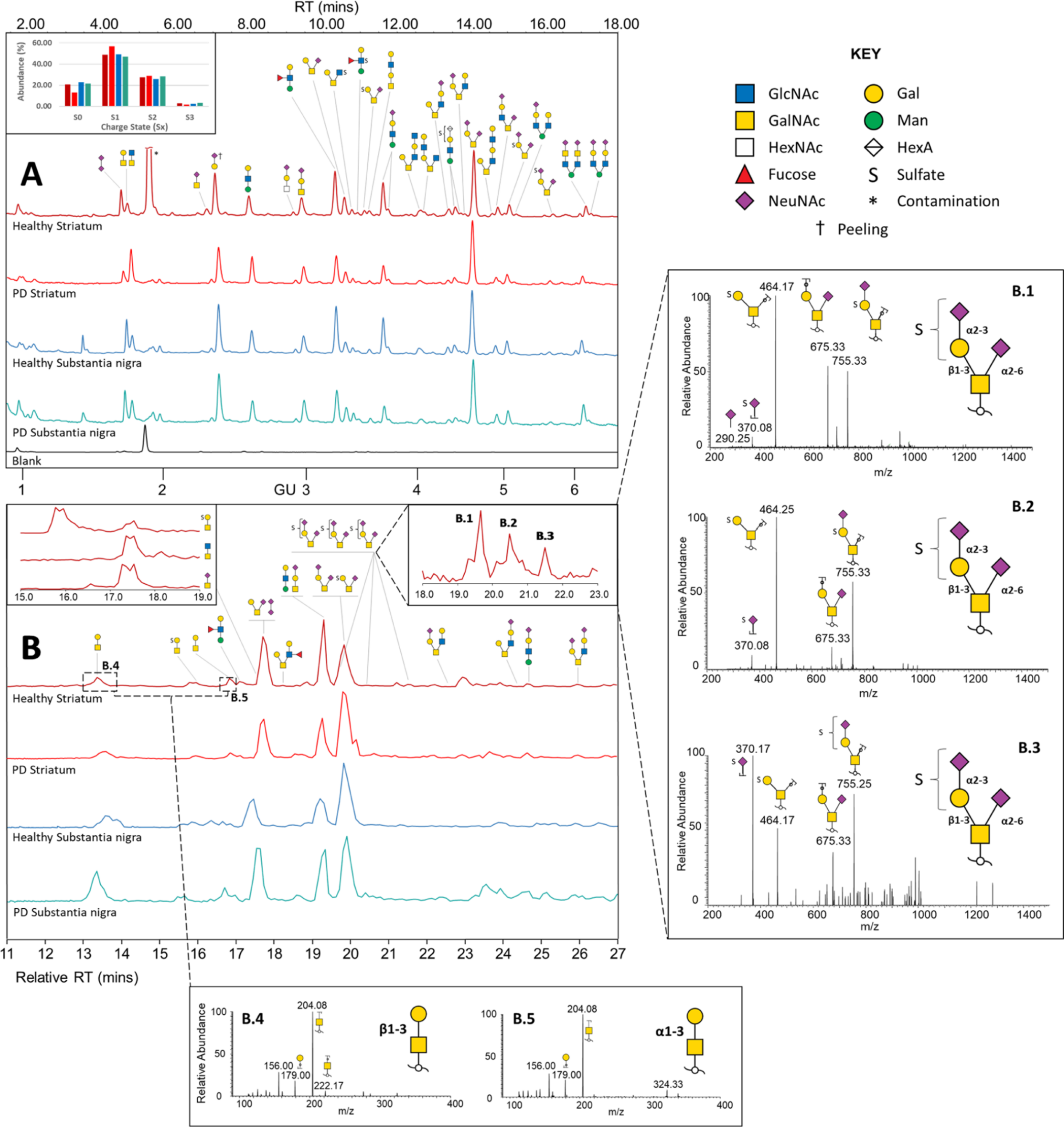
(A) Representative FLR-HILIC-UPLC chromatograms (top) of *O*-glycans in brain sample pools. Integrated peaks on chromatograms are shown in Figure S4. The inset FLR-WAX-UPLC graph shows relative abundance of 2AB-labeled glycans by the charge state (S*x*) (bar colors correspond to chromatogram colors). FLR-WAX-UPLC chromatograms shown in Figure S5. (B) PGC-LC-MS^*n*^ base peak chromatograms (bottom) of *O*-glycans in brain sample pools (relative RT to the healthy striatum). Insets show example EICs outlining coelution of structures (left; *m*/*z* 464, 425, and 513) and separation of glycan isomers (right; *m*/*z* 1046—B.1–B.3). Further MS^2^ spectra of these three isomers are shown on the right panel, indicating structures which place the sulfate modification on the galactose or the sialic acid residue, outlining the circumstance of potential sulfate migration during MS detection. Core 1 and proposed core 8 *O*-glycan alditol isomers (*m*/*z* 384) analyzed with PGC-LC-MS^2^ are shown in (B.4,B5). The elution time of Galβ1-3GalNAc at 13.4 min was confirmed using reference standards analyzed at the same occasion. Only major structures are shown.

Our detailed structural analyses uncovered 70 *O*-glycans covering GalNAc and mannose cores (Table S4). A total of 49 glycans were the more common, mucin-type GalNAc core glycans, and 21 were assigned as mannose-core structures based on previous knowledge of mammalian mannose-core glycans in the brain.^24,37^ In addition, there were two confirmed peeling products (Gal and NeuNAcα2-3Gal) and two more possible peeling products containing a HexNAc reducing end [Fucα1-2Galβ1-3(Fucα1-4)HexNAc and NeuNAcα2-6Galβ1-3HexNAc], which do not typify common GalNAc core structures and may instead contain GlcNAc at their core. A disialic structure (NeuNAc–NeuNAc) was also detected, which may have peeled from larger unconfirmed structures. Exoglycosidase panels used to characterize the assigned glycan structures are illustrated in Tables S5–S8, including determination of the monosaccharide elution order (Figure S2), and UPLC and mass spectrometry data are presented in Table S9, including shorthand names for *O*-glycans (Table S10). The chromatograms in Figure 2 show major *O*-glycans from the striatum and substantia nigra from healthy and controls and outline how detection sensitivity of glycans varies by the two different methods, such as charged species which ionize more readily than neutral in negative-ion MS and may appear over-represented. Figure 2A also shows the stability of RTs in FLR-HILIC-UPLC across sample runs. The ability of PGC-HPLC (Figure 2B) to separate glycan isomers and the ability to mine specific ions using parent extracted ion chromatograms (EICs) and MS^2^ fragmentation data make this combination of methods particularly powerful.

Glycan isomers with varying monosaccharide and sulfate linkage positions were identified, for example, sulfated disialyl T-antigen structures identified by MS^2^ in Figure 2 and further characterized by exoglycosidase and MS of labeled glycans (Figure S3), as well as the isomers of the sialylated mannose-core tetrasaccharide (peak 25–26 in FLR-HILIC-UPLC). Of the latter, structural isomers containing terminal α2-3 and α2-6 linked sialic acids on both β1-3 and β1-4 galactosylated structures were identified through digestion with linkage-specific sialidases (Figure S6) changing the associated integrated peak areas (Table S2).

The digestion profiles of two uncommon structures NeuNAcα2-6-(Fucα1-2Galβ1-3)[GlcNAcβ1-6]GalNAc and Galβ1-3[Galβ1-3GalNAcβ1-4GlcNAcβ1-6]GalNAc were investigated (Figure S7). While these glycans are at low abundance compared to dominant peaks (<0.5% total chromatogram area) and may appear incommensurable to major peaks, their peak area shifts from digestions give an indication to their conformation (coupled with exoglycosidase panel calculations for healthy and PD substantia nigra, respectively; Tables S7–S8). In the case of 2AB-labeled sulfated glycans that resist digestion, FLR-HILIC-UPLC-MS spectra/chromatograms were compared directly with offline FLR-HILIC-UPLC chromatograms to confirm their GU/elution position and referenced against the PGC-LC-MS^*n*^ data of their reduced alditol equivalent to determine the structure. An example is shown in the case of two identified sulfo-hexuronylated structures [confirmed with MS and proposed through literature analysis of mannose-core *O*-glycans to be *sulfo*-*glucuronylated* “human natural killer-1”-epitope-tetrasaccharides—[M – H]^−^ ion of *m*/*z* 920 for 2AB-labeled and *m*/*z* 802 for reduced alditol (Figure S8)]. Two noteworthy structural isomers confirmed with PGC separation and MS^2^ analysis of glycan alditols consisted of a Hex and a reduced HexNAc and were found in all four brain tissue samples. The first peak was interpreted as the mucin-type core 1 T-antigen (Galβ1-3GalNAc) by comparing the MS^2^ and RT to reference samples (unicarb.db). The second disaccharide eluted approximately 3 min later and revealed identical MS^2^ spectra and was proposed to correspond to the core 8 disaccharide Galα1-3GalNAc^38^ [Figure 2(B.5)]. Other glycans of interest analyzed with PGC-LC-MS^*n*^ are outlined in the spectra in Figure S9.

### *O*-Glycome Changes in PD and ILBD

**Figure 3:**
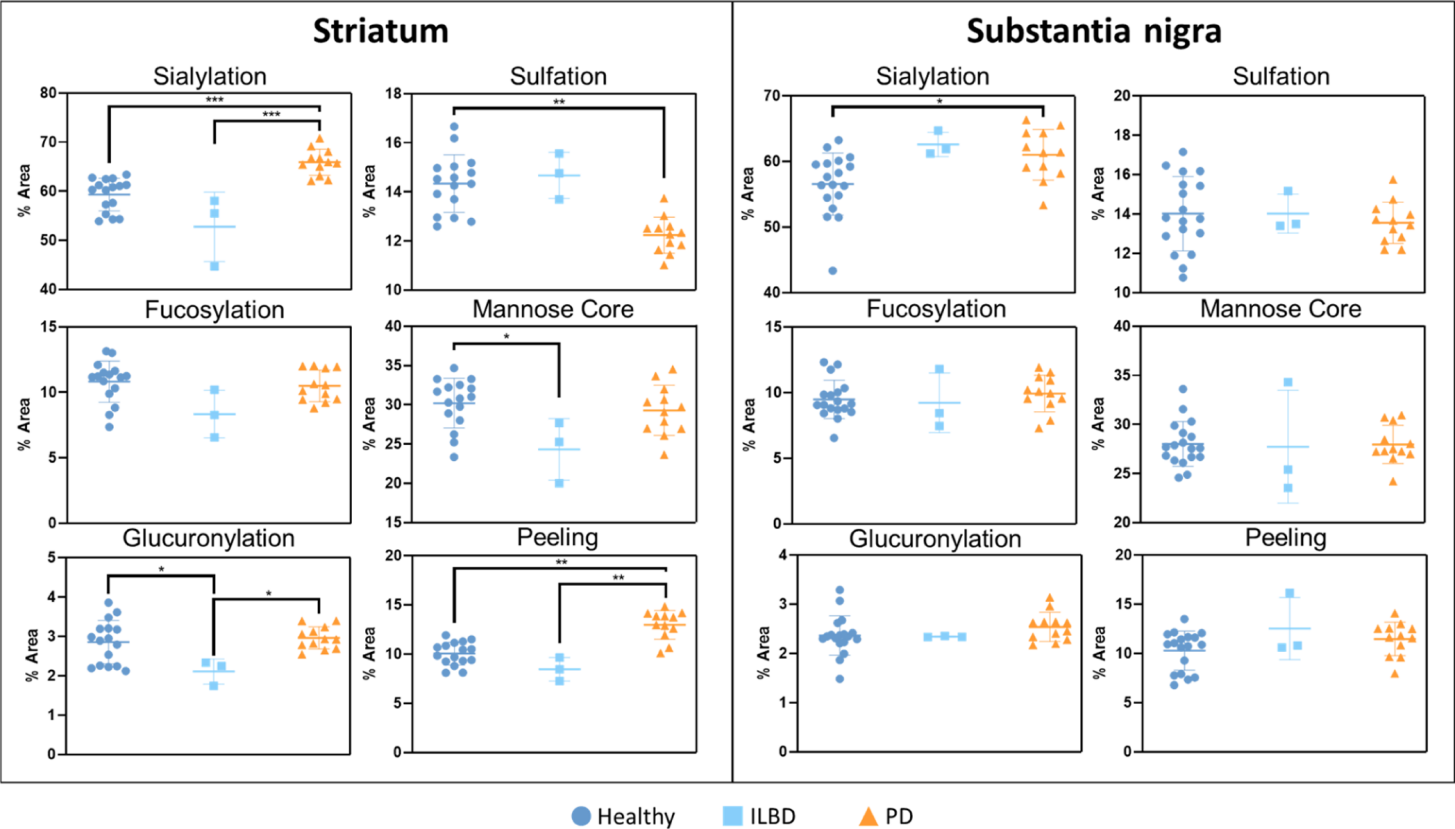
Nested plots of significant changes in striatum and substantia nigra based on grouping of identified glycans by feature (sialylation, fucosylation, sulfation, glucuronylation, glycans with mannose cores, and peeling). % areas for these features in the individual samples in each group were compared against disease states in the striatum and substantia nigra. The significance across groups is written as follows: * (*p* < 0.05), ** (*p* < 0.01), and *** (*p* < 0.001).

Samples were divided into six groups to identify statistical significance: healthy striatum, ILBD (Braak stage 1–2) striatum, PD (Braak stage 3–4) striatum, healthy substantia nigra, ILBD substantia nigra, and PD substantia nigra. Nested plots of significant changes in striatum and substantia nigra are shown in Figure 3 based on grouping of identified glycans by feature. Complete tables of *p*-values for individual peak and feature differences are shown in Tables 1 and 2.

**Table 1:**
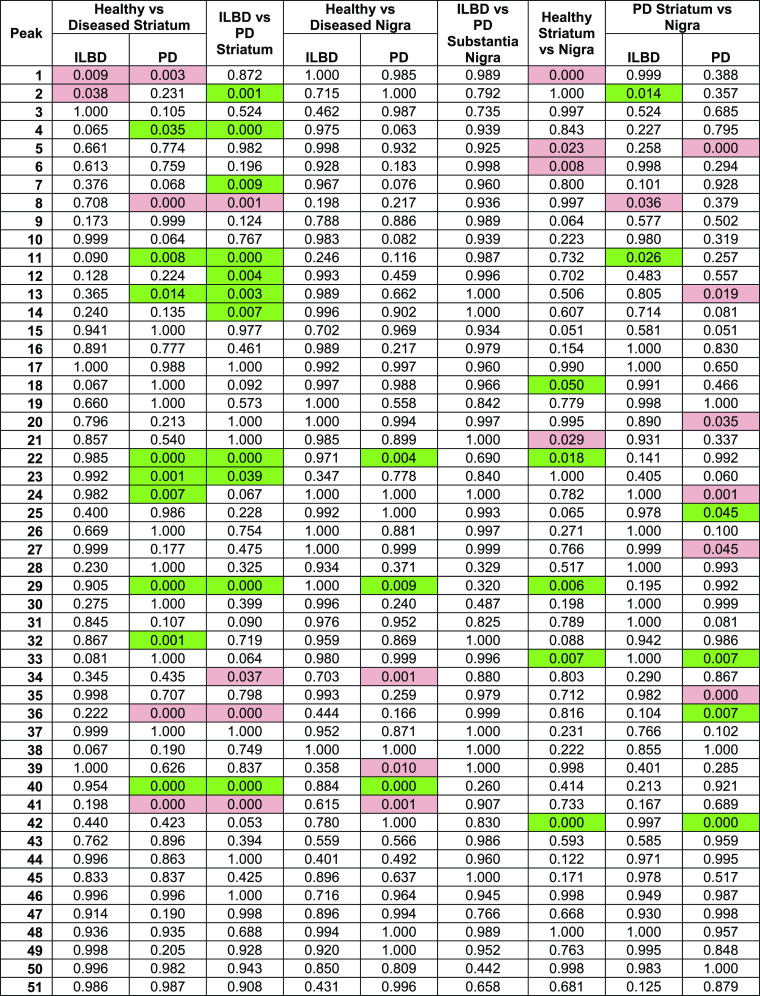
*p*-Values Derived from Comparison of Each Peak across Different Groups Comparing Disease States^a^

a Red indicates a decrease in relative abundance in the leading group (*e.g.*, the healthy group in healthy *vs* PD), and green indicates an increase—only statistically significant areas highlighted.

**Table 2:**
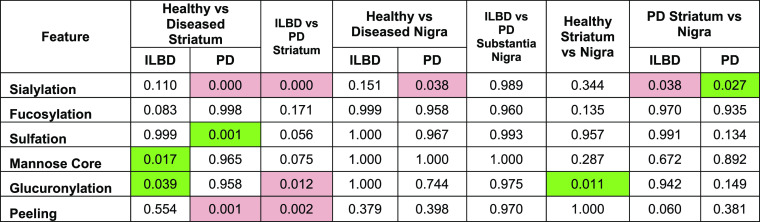
*p*-Values Derived from Comparison of Glycan Features across Different Groups Comparing Disease States^a^

a Red indicates a decrease in relative abundance in the leading group (*e.g.*, the healthy group in healthy *vs* PD), and green indicates an increase—only statistically significant areas highlighted. Main glycan of each peak used for analysis.

Total mannose-core glycans (*p* = 0.017) and total glucuronylation (*p* = 0.039) were significantly decreased in the ILBD striatum compared with healthy controls, although no individual glycan was significantly different.

*At the later stages of PD pathology* (Braak stages 3–4), the *striatum* expressed an increase in the overall sialylation (*p* = 0.000), driven by patients over 85 years of age (*p* = 0.008; Table S11), and a decrease in total sulfation (*p* = 0.001), driven by females (*p* = 0.022 in females compared to *p* = 0.305 in males; Tables S12 and S13, respectively). To note is the significant increase in the overall peeling (*p* = 0.001). While not truly representative of any physiological glycosylation *in vivo*, there is some value gained from this unwanted product. The majority of this peeling feature (>90%) is derived from the glycan product NeuNAcα2-3Gal (peak 8). Despite peeling being an unwanted side reaction, the sialylation of this structure contributes to the calculation of the overall sialylation, as it originates most likely from larger mucin-type sialylated *O*-glycan structures. Including this peeling product, the disialyl t-antigen (peak 36) and its sulfated analogue SO_3_-NeuNAcα2-3Galβ1-3[NeuNAcα2-6]GalNAc “isomer 2” (peak 41; unidentified sulfation position on the galactose/sialic acid, so it is differentiated by the isomer number) were all significantly increased in PD (all with *p* < 0.001), while HSO_3_-NeuNAcα2-6GalNAc (peak 4; *p* = 0.035), HSO_3_-Galβ1-3[NeuNAcα2-6]GalNAc (peak 22; *p* < 0.001), Galβ1-3[NeuNAcα2-3Galβ1-4GlcNAcβ1-6]GalNAc (peak 32; *p* = 0.001), and HSO_3_-NeuNAcα2-3Galβ1-3[NeuNAcα2-6]GalNAc “isomer 1” (peak 40; *p* = 0.000) were decreased. These up- and downregulated sulfated structures are the same structures that contribute to significant sulfation changes.

*By comparing ILBD to the PD striatum*, there were similar changes observed in healthy versus PD striatum (sialylation and peeling increased in PD), with the addition of increased glucuronylation in PD (*p* = 0.012) coming from the significant increase in SO_3_-GlcAβ1-3Galβ1-3GlcNAcβ1-2Man (peak 34; *p* = 0.037). While sialylation remained significantly increased in PD (*p* < 0.001), there was a notable decrease in abundance of the sialyl-Tn antigen (peak 7; *p* = 0.009).

Interestingly, little significant difference was observed in any *O*-glycosylation features between healthy, ILBD, or PD substantia nigra, except for an ***increase in the overall sialylation*** (*p* = 0.038) ***in PD substantia nigra***, driven by males (*p* = 0.030 in males compared to *p* = 0.987 in females; Tables S13 and S12, respectively). This was attributed to an increase in NeuNAcα2-3Galβ1-3GlcNAcβ1-3[NeuNAcα2-6]GalNAc (peak 39; *p* = 0.010) and HSO_3_-NeuNAcα2-3Galβ1-3[NeuNAcα2-6]GalNAc “isomer 1” (peak 40, *p* = 0.001) in the PD group and a decrease in HSO_3_-Galβ1-3[NeuNAcα2-6]GalNAc (peak 22; *p* = 0.004) and HSO_3_-NeuNAcα2-3Galβ1-3[NeuNAcα2-6]GalNAc “isomer 2” (peak 41; *p* = 0.000). There was, however, no significant difference in any feature when comparing ILBD and PD substantia nigra.

**Figure 4:**
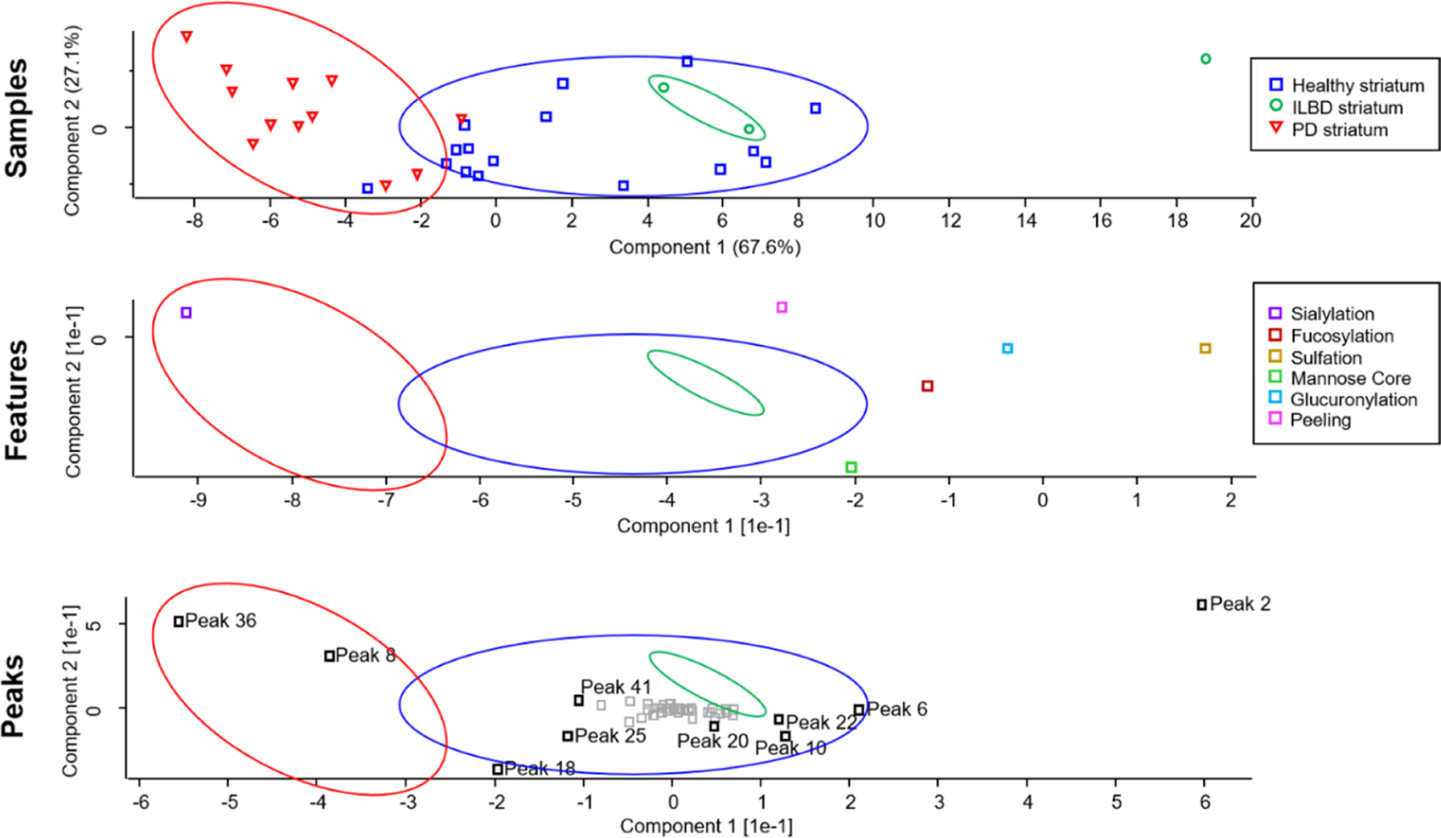
PCA plots outlining separation (difference) of the healthy striatum, ILBD striatum, and PD striatum. Grouped areas were superimposed over feature and peak PCA plots to identify components that drive the difference.

By plotting peaks and feature values for each sample using PCA which represents likeness between different results (where results within a cluster are the most similar and clusters in separate regions of the plot are the most different), it was determined that sialylation was the main component separating healthy and PD striata (derived from peaks 36; sialyl T-antigen and peak 8; NeuNAcα2-3Gal—Figure 4), emphasizing a difference with more granularity over *p*-value readouts from MANOVA analyses (Tables 1 and 2). The overlap, or lack of separation, of healthy and ILBD striatum samples indicates that ILBD and the healthy control share a lot of similarities (peak areas and features); however, this could be attributed to the small sample set for ILBD (*n* = 3). Peaks 6 (core 1, core 8, and core 3 disaccharides), 10 (Galβ1-4GlcNAcβ1-2Man), 18 [Galβ1-4(Fucα1-3)GlcNAcβ1-2Man], 20 (Galβ1-3[HSO_3_-GlcNAcβ1-6]GalNAc), 22 (HSO_3_-Galβ1-3[NeuNAcα2-6]GalNAc), 25 (NeuNAcα2-3Galβ1-4GlcNAcβ1-2Man), and 41 (HSO_3_-NeuNAcα2-3Galβ1-3[NeuNAcα2-6]GalNAc “isomer 2”) can be seen to be attributed to differences in the healthy striatum. These results match closely the results obtained from MANOVA analysis with respect to sialylated glycan signatures in the PD striatum; however, PCA was able to emphasize a more noticeable difference in the sialyl T-antigen (peak 36) and NeuNAcα2-3Gal (peak 8) glycan peaks between healthy and PD (Figure 4) compared with MANOVA analysis which also considered HSO_3_-NeuNAcα2-3Galβ1-3[NeuNAcα2-6]GalNAc “isomer 2” (peak 41) to be equally significant (all three peaks were equally significant; *p* = 0.000—Table 1). Complete PCA plots for all comparisons can be seen in Figures S10–S19.

### Temporal and Spatial Differences in the Healthy, ILBD, and PD Striatum and Substantia Nigra

Comparing spatially separated healthy controls, there were significantly higher relative abundances of glucuronylation (*p* = 0.011) in the healthy striatum compared with substantia nigra. With respect to the significance of individual glucuronic acid-containing structures, HSO_3_-GlcAβ1-3Galβ1-4GlcNAcβ1-2Man (peak 33; *p* = 0.007) was at a higher abundance in the healthy striatum. When observing temporally the glycosylation of both regions after the onset of ILBD, there were higher levels of sialylation in substantia nigra compared with the striatum indicated by an increase in NeuNAcα2-3Gal (peak 8; *p* = 0.036); however, upon the onset of later Braak stage 3–4 PD in both groups, sialylation (*p* = 0.15) was significantly higher in the striatum. Significant individual glycan differences driving higher levels of sialyation for the PD striatum came from increases in NeuNAcα2-3Galβ1-4GlcNAcβ1-2Man (peak 25; *p* = 0.045), disialyl t-antigen (peak 36; *p* = 0.007), and NeuNAcα2-3Galβ1-4GlcNAcβ1-2[Galβ1-4GlcNAcβ1-6]Man (peak 42; *p* = 0.000); however, decreases in NeuNAc–NeuNAc (peak 5; *p* = 0.000), Galβ1-3[NeuNAcα2-6]GalNAc (peak 27; *p* = 0.045), and NeuNAcα2-3Galβ1-3[Galβ1-4GlcNAcβ1-6]GalNAc (peak 35; *p* = 0.000) were also seen.

## Discussion

Making up approximately 30% of the total glycome in each group, ***mannose-core glycans*** have been linked to CMDs such as Walker–Warburg syndrome, Fukuyama CMD, and CMD 1C/1D, with underglycosylation of these structures on α-dystroglycan inhibiting this cell adhesion molecule from correctly binding extracellular matrix proteins.^39,40^ While its link to ILBD and PD is ambiguous in the literature, the downregulation of mannose-core glycans in the ILBD striatum may help outline the possible causes for any dystrophic changes that may be seen in ILBD patients. Additionally, *O*-mannosyl glycans have been suggested to act as a scaffold for Lewis X epitopes (identified across a number of structures in this work) which are an important feature highly expressed in the developing brain^41^ and have been shown to produce anxiety-like behavior in mutant mice lacking the α1-3 fucosyltransferase IX.^42^***Glucuronylation*** has been included in feature analysis due to its unique nature in typical *O*-glycome analyses of mucin-type samples, as well as the fact that it plays a vital role in cell–cell and cell–matrix neuronal adhesion, and in performing functions associated with nervous system development.^40,43,44^ The presence of sulfated glucuronic acid epitope at the end of the mannose-core glycans (called the human natural killer-1 antigen) in this project (two glycans: HSO_3_-GlcAβ1-3**Galβ1-4**GlcNAcβ1-2Man and HSO_3_-GlcAβ1-3Gal**β1-3**GlcNAcβ1-2Man) gives an indication to the degree of glucuronylation, or more specifically, sulfoglucuronylation. As these structures are important carriers for functional epitopes, the apparent downregulation of this feature in ILBD provides an interesting insight into its potential role in neurodegeneration; however, more work will need to be carried out to clarify its importance.

In contrast to previous work conducted on PD *N*-glycosylation where it was found that sialylation of *N*-glycans was decreased in PD patients’ IgG,^22^***sialylation*** of *O*-glycans appeared to increase in the striatum of PD patients. Furthermore, the degree of sialylation is known to mediate specific receptor interactions with sialic acid binding ligands such as siglecs and selectins which inhibit complement components binding to cell surfaces, inducing complement-mediated proinflammatory cascades.^45^ This interesting contrast observed for *O*-glycans will need to be investigated in future work to identify the difference. However, one important observation made across MANOVA and PCA analyses was that the sialylation increase in the striatum of PD patients was driven by an increase in NeuNAcα2-3Galβ1-3[NeuNAcα2-6]GalNAc. This disialylated structure has been associated with various forms of cancer,^46^ and the increase in α2-6-linked sialic acid-containing structures like this one has also been correlated to an enhanced degenerative state in the brain.^47^ Similar outcomes were observed in other neurodegenerative diseases such as Alzheimer’s disease (AD), where sialylation events occurring in the serum *N*-glycome matched that in PD. However, the total *O*-glycome of AD seems yet to be elucidated also. By observing changes in PD substantia nigra, sialylation was increased relative to healthy control, and the core 3-extended structure NeuNAcα2-3Galβ1-3GlcNAcβ 1–3[NeuNAcα2-6]GalNAc and a sulfated version of the disialylated core 1 structure observed in the PD striatum (HSO_3_-NeuNAcα2-3Galβ1-3[NeuNAcα2-6]GalNAc) were upregulated. The hypothesis of increased 6-linked sialic acid-associated neurodegeneration is reinforced by these results. Furthermore, the male-driven sialylation increases in PD substantia nigra seen in this work align with previous reports on serum *N*-glycosylation where more significant sialylation alterations were observed in males.^21^

Regarding ***sulfation***, studies have determined that sulfotransferase deficiency is linked to abnormal myelination and axonal degeneration of the peripheral nervous system (*N*-glycosylation experiment in mice).^48^ This axonal demyelination causes permanent neurological disability seen in human myelin diseases.^48^ Again, while studies on sulfation of *N*- and *O*-glycans are limited in the literature with respect to PD, the similarities here, of decrease in sulfation in the PD striatum compared with the healthy control, is worth investigating in future work.

While ILBD expressed an upregulation of mannose-core and glucuronylated structures, the overall differences between ILBD and healthy controls according to PCA analyses were minimal, indicating that the groups were similar, compared against PD. This is somewhat expected. As discussed, ILBD and Lewy body formation had an association with aging rather than PD. Furthermore, according to Braak staging, substantia nigra regions remain unaffected in ILBD (stage 1 and 2) and only affect this region in the later stage (3 and 4) patients. This corroborates the difficulty in acquiring brain samples from ILBD patients (and the low n number used in this study), since these patients would not be diagnosed with PD, so they would only be considered “healthy” until postmortem analysis. The other main observation was that even at later stage PD, the striatum appeared more affected than substantia nigra brain regions. While significant alterations in *O*-GlcNAcylation of α-synuclein in substantia nigra have been heavily analyzed and reviewed and contribute significantly to Lewy body formation and neuronal degradation in the substantia nigra,^12^ alterations in the *total O*-glycome of this region do not follow this pattern; substantia nigra *O*-glycome change is limited compared to controls. This indicates that future biomarker identification and regenerative therapeutic targets for *O*-glycans in PD patients may be benefitted by observing solely the striatum, where significant glycosylation changes occur and where most regenerative therapeutic treatments for PD target occur.^49,50^ Also observed, the striatum and substantia nigra share much the same glycan structures, albeit at different relative abundances. Of all features, only glucuronylation was different between the healthy control striatum (higher relative abundance) and substantia nigra. In ILBD comparisons between regions, sialylation was higher in substantia nigra; however, in PD comparisons, sialylation was higher in the striatum. Due to their nigrostriatal connection and proximity in the brain, the similarities are not unexpected.

In addition to biomarker identification for these diseases, the presence of the core 8 disaccharide provides an interesting insight into *O*-glycosylation in brain tissue glycoproteins as this feature appears far more restrictive compared to its core 1 analogue, limitedly reported in human respiratory mucin using NMR studies.^51^ In addition, a study of *O*-glycans purified from rat brain tissue in 1975 reported on equal amounts of core 8 and the core 1 disaccharide as observed by Finne.^38^ The identification of this rare core 8 glycan in brain cross species points toward a very specific and important function of this oligosaccharide in mammals. As this α-galactose epitope is typically immunogenic in humans, it and its associated glycosyltransferase in the brain hint at the extreme efficiency of the blood–brain barrier in preventing immunoglobulins such as the anti-Gal antibody to pass.^52,53^

Additional analytical considerations outside the direct scope of this report can be found in the “Supporting Discussion” section of the “Supporting Information” document (pages S-3–S-5).

## Conclusions

To our knowledge, this exploratory study of the *O*-glycome of nigrostriatal tissues in human PD and ILBD is the first of its kind using novel glycan release approaches alongside traditional techniques, outlining the heterogeneity of *O*-glycan structures found in these unexplored regions. The combinatorial analytical approach using well-established reductive release methodologies alongside this optimized, low-peeling microwave-assisted nonreductive release permitted incorporation of MS techniques associated with typical reduced *O*-glycan analysis, as well as exoglycosidase practices (typically limited to *N*-glycan workflows) allowing identification and meaningful statistical evaluation. Potential biomarkers have been identified that may, in future, be targets in the development of glyco-therapeutic treatments for these diseases. We hope, in addition to further exploratory testing using other orthogonal methods (including blind analysis testing which was not performed), that this work will help in detailing pathogenesis of PD, as well as other similar neurodegenerative diseases.

## Supporting Information Available

Additional discussion outlining analytical considerations for characterization and potential improvements for the nonreductive *O*-glycan release method; development of a microwave-assisted nonreductive β-elimination *O*-glycan release of fetuin and porcine gastric mucin standard glycoproteins; monosaccharide elution order from exoglycosidase digestions of *O*-glycans from PD substantia nigra, released with nonreductive release and analyzed as 2AB-labeled derivatives with FLR-HILIC-UPLC; representative FLR-HILIC-UPLC chromatograms of healthy/PD/ILBD striatum and substantia nigra *O*-glycans; FLR-WAX-UPLC chromatograms of nonreductively released 2AB-labeled *O*-glycans from standard and test samples; offline exoglycosidase chromatograms of nonreductively released 2AB-labeled *O*-glycans from PD substantia nigra pool alongside EICs and online FLR chromatogram of FLR-HILIC-UPLC-ESI-MS analysis; digestion profile from FLR-HILIC-UPLC of sialylated mannose-core tetrasaccharide isomers from the healthy striatum; glycosidase digestion profiles of two 2AB-labeled *O*-glycans from healthy and PD substantia nigra; comparing 2AB-labeled sulfated glycans (HexA-Gal-GlcNAc-Man) from FLR-HILIC-UPLC-MS spectra to offline FLR-HILIC-UPLC profiles; EICs and MS^2^ spectra of selected *O*-glycans; PCA comparing glycan features of individual samples in healthy control, PD, and ILDB substantia nigra tissues; PCA comparing glycan peaks of individual samples in healthy control, PD, and ILDB substantia nigra tissues; PCA comparing glycan features between individual samples in the healthy striatum and substantia nigra tissues; PCA comparing glycan peaks between individual samples in the healthy striatum and substantia nigra tissues; PCA comparing glycan features between individual samples in the ILBD striatum and substantia nigra tissues; PCA comparing glycan peaks between individual samples in the ILBD striatum and substantia nigra tissues; PCA comparing glycan features between individual samples in the PD striatum and substantia nigra tissues; PCA comparing glycan peaks between individual samples in the PD striatum and substantia nigra tissues; PCA comparing glycan features between individual samples in the ILBD and PD (pooled together) striatum and substantia nigra tissues; PCA comparing glycan peaks between individual samples in the ILBD and PD (pooled together) striatum and substantia nigra tissues; details of the human brain tissue of PD, ILBD, and control cases; % areas of major fetuin *O*-glycan structures for all parameters tested when developing the microwave release method; CV calculation for 10 major peaks in the brain tissue nonreductive glycan release reproducibility test; *O*-glycans characterized across all PD and control groups; exoglycosidase digestion panel for the healthy striatum pool (refer to Supporting Spreadsheet); exoglycosidase digestion panel for the PD (stages 1–4) striatum pool (refer to Supporting Spreadsheet); exoglycosidase digestion panel for healthy substantia nigra pool (refer to Supporting Spreadsheet); exoglycosidase digestion panel for the PD (stages 1–4) substantia nigra pool (refer to Supporting Spreadsheet); PGC-LC-MS, FLR-HILIC-UPLC-MS, and FLR-HILIC-UPLC data for reduced and nonreduced glycans (refer to Supporting Spreadsheet); short-hand naming convention of *O*-glycans used in the Tables S5–S9; *p*-values derived from a comparison of glycan features across different groups comparing disease states separated by age (“under 85” and “85+”); *p*-values derived from comparison of glycan features across different groups comparing disease states from females; and *p*-values derived from comparison of glycan features across different groups comparing disease states from males (PDF)
Larger data sets that were unsuitable for condensing into the “supporting tables” section (XLSX)

## Notes

Data Deposition: raw MS data of reduced *O*-glycans and assigned glycomics structures are available at https://glycopost.glycosmos.org/ using the project ID: GPST000185.^54^ Structures with the available MIRAGE information are available at Unicarb-DR (https://unicarb-dr.glycosmos.org/references/449). Raw MS data of nonreduced *O*-glycans and assigned glycomics structures are available at https://glycopost.glycosmos.org/ using the project ID: GPST000195.^54^

## References

[ref1] Ghazarian H.; Idoni B.; Oppenheimer S. B. A glycobiology review: carbohydrates, lectins and implications in cancer therapeutics. Acta Histochem. 2011, 113, 236–247. 10.1016/j.acthis.2010.02.004. 20199800 PMC3027850

[ref2] Lan Y.; Hao C.; Zeng X.; He Y.; Zeng P.; Guo Z.; Zhang L. Serum glycoprotein-derived N- and O-linked glycans as cancer biomarkers. Am. J. Cancer Res. 2016, 6, 2390–2415. 27904760 PMC5126262

[ref3] Varki A.; Cummings R. D.; Esko J. D.; Stanley P.; Hart G. W.; Aebi M.; Darvill A.; Kinoshita T.; Packer N. H.; Prestegard J. H.; Schnaar R. L.; Seeberger P. H. Essentials of Glycobiology [Internet], 3rd ed.; Cold Spring Harbor Laboratory Press: Cold Spring Harbor, NY, 2015–2017; Chapter 50. 27010055

[ref4] Wilkinson H.; Saldova R. Current Methods for the Characterization of O-Glycans. J. Proteome Res. 2020, 19, 3890–3905. 10.1021/acs.jproteome.0c00435. 32893643

[ref5] Kozak R. P.; Royle L.; Gardner R. A.; Fernandes D. L.; Wuhrer M. Suppression of peeling during the release of O-glycans by hydrazinolysis. Anal. Biochem. 2012, 423, 119–128. 10.1016/j.ab.2012.01.002. 22306471

[ref6] Maniatis S.; Zhou H.; Reinhold V. Rapid de-O-glycosylation concomitant with peptide labeling using microwave radiation and an alkyl amine base. Anal. Chem. 2010, 82, 2421–2425. 10.1021/ac902734w. 20178317 PMC2837759

[ref7] Zhao S.; Walsh I.; Abrahams J. L.; Royle L.; Nguyen-Khuong T.; Spencer D.; Fernandes D. L.; Packer N. H.; Rudd P. M.; Campbell M. P. GlycoStore: a database of retention properties for glycan analysis. Bioinformatics 2018, 34, 3231–3232. 10.1093/bioinformatics/bty319. 29897488

[ref8] Royle L.; Mattu T. S.; Hart E.; Langridge J. I.; Merry A. H.; Murphy N.; Harvey D. J.; Dwek R. A.; Rudd P. M. An analytical and structural database provides a strategy for sequencing O-glycans from microgram quantities of glycoproteins. Anal. Biochem. 2002, 304, 70–90. 10.1006/abio.2002.5619. 11969191

[ref9] Leymarie N.; Zaia J. Effective use of mass spectrometry for glycan and glycopeptide structural analysis. Anal. Chem. 2012, 84, 3040–3048. 10.1021/ac3000573. 22360375 PMC3319649

[ref10] Morelle W.; Faid V.; Chirat F.; Michalski J.-C. Analysis of N- and O-linked glycans from glycoproteins using MALDI-TOF mass spectrometry. In Glycomics: Methods and Protocols; Packer N. H., Karlsson N. G., Eds.; Humana Press: Totowa, NJ, 2009; pp 3–21. 10.1007/978-1-59745-022-5_1 19277556

[ref11] Politis M.; Wu K.; Molloy S.; Bain P. G.; Chaudhuri K. R.; Piccini P. Parkinson’s disease symptoms: the patient’s perspective. Mov. Disord. 2010, 25, 1646–1651. 10.1002/mds.23135. 20629164

[ref12] Videira P. A. Q.; Castro-Caldas M. Linking glycation and glycosylation with inflammation and mitochondrial dysfunction in Parkinson’s disease. Front. Neurosci. 2018, 12, 381 10.3389/fnins.2018.00381. 29930494 PMC5999786

[ref13] Dauer W.; Przedborski S. Parkinson’s disease: mechanisms and models. Neuron 2003, 39, 889–909. 10.1016/s0896-6273(03)00568-3. 12971891

[ref14] Nowak P.; Kostrzewa R. M.; Kwieciński A.; Bortel A.; Labus Ł.; Brus R. Neurotoxic action of 6-hydroxydopamine on the nigrostriatal dopaminergic pathway in rats sensitized with D-amphetamine. J. Physiol. Pharmacol. 2005, 56, 325–333. 15985712

[ref15] Kim W. S.; Kågedal K.; Halliday G. M. Alpha-synuclein biology in Lewy body diseases. Alzheimer’s Res. Ther. 2014, 6, 73 10.1186/s13195-014-0073-2. 25580161 PMC4288216

[ref16] Lashuel H. A.; Overk C. R.; Oueslati A.; Masliah E. The many faces of α-synuclein: from structure and toxicity to therapeutic target. Nat. Rev. Neurosci. 2013, 14, 38–48. 10.1038/nrn3406. 23254192 PMC4295774

[ref17] Braak H.; Tredici K. D.; Rüb U.; de Vos R. A. I.; Jansen Steur E. N. H.; Braak E. Staging of brain pathology related to sporadic Parkinson’s disease. Neurobiol. Aging 2003, 24, 197–211. 10.1016/s0197-4580(02)00065-9. 12498954

[ref18] Dickson D. W.; Fujishiro H.; DelleDonne A.; Menke J.; Ahmed Z.; Klos K. J.; Josephs K. A.; Frigerio R.; Burnett M.; Parisi J. E.; Ahlskog J. E. Evidence that incidental Lewy body disease is pre-symptomatic Parkinson’s disease. Acta Neuropathol. 2008, 115, 437–444. 10.1007/s00401-008-0345-7. 18264713

[ref19] DelleDonne A.; Klos K. J.; Fujishiro H.; Ahmed Z.; Parisi J. E.; Josephs K. A.; Frigerio R.; Burnett M.; Wszolek Z. K.; Uitti R. J.; Ahlskog J. E.; Dickson D. W. Incidental Lewy body disease and preclinical Parkinson disease. Arch. Neurol. 2008, 65, 1074–1080. 10.1001/archneur.65.8.1074. 18695057

[ref20] Frigerio R.; Fujishiro H.; Ahn T.-B.; Josephs K. A.; Maraganore D. M.; DelleDonne A.; Parisi J. E.; Klos K. J.; Boeve B. F.; Dickson D. W.; Ahlskog J. E. Incidental Lewy body disease: do some cases represent a preclinical stage of dementia with Lewy bodies?. Neurobiol. Aging 2011, 32, 857–863. 10.1016/j.neurobiolaging.2009.05.019. 19560232 PMC3366193

[ref21] Váradi C.; Nehéz K.; Hornyák O.; Viskolcz B.; Bones J. Serum N-glycosylation in Parkinson’s disease: a novel approach for potential alterations. Molecules 2019, 24, 2220 10.3390/molecules24122220. PMC6630595 31200590

[ref22] Russell A. C.; Šimurina M.; Garcia M. T.; Novokmet M.; Wang Y.; Rudan I.; Campbell H.; Lauc G.; Thomas M. G.; Wang W. The N-glycosylation of immunoglobulin G as a novel biomarker of Parkinson’s disease. Glycobiology 2017, 27, 501–510. 10.1093/glycob/cwx022. 28334832

[ref23] Marotta N. P.; Lin Y. H.; Lewis Y. E.; Ambroso M. R.; Zaro B. W.; Roth M. T.; Arnold D. B.; Langen R.; Pratt M. R. O-GlcNAc modification blocks the aggregation and toxicity of the protein α-synuclein associated with Parkinson’s disease. Nat. Chem. 2015, 7, 913–920. 10.1038/nchem.2361. 26492012 PMC4618406

[ref24] Meng C.; Sasmal A.; Zhang Y.; Gao T.; Liu C.-C.; Khan N.; Varki A.; Wang F.; Cao H. Chemoenzymatic assembly of mammalian O-mannose glycans. Angew. Chem., Int. Ed. 2018, 57, 9003–9007. 10.1002/anie.201804373. PMC6176721 29802667

[ref25] Breloy I.; Pacharra S.; Aust C.; Hanisch F.-G. A sensitive gel-based global O-glycomics approach reveals high levels of mannosyl glycans in the high mass region of the mouse brain proteome. Biol. Chem. 2012, 393, 709–717. 10.1515/hsz-2012-0214. 22944674

[ref26] Sheikh M. O.; Halmo S. M.; Wells L. Recent advancements in understanding mammalian O-mannosylation. Glycobiology 2017, 27, 806–819. 10.1093/glycob/cwx062. 28810660 PMC6082599

[ref27] Ma L.; Song J.; Sun X.; Ding W.; Fan K.; Qi M.; Xu Y.; Zhang W. Role of microtubule-associated protein 6 glycosylated with Gal-(β-1,3)-GalNAc in Parkinson’s disease. Aging 2019, 11, 4597–4610. 10.18632/aging.102072. 31289257 PMC6660046

[ref28] Royle L.; Campbell M. P.; Radcliffe C. M.; White D. M.; Harvey D. J.; Abrahams J. L.; Kim Y.-G.; Henry G. W.; Shadick N. A.; Weinblatt M. E.; Lee D. M.; Rudd P. M.; Dwek R. A. HPLC-based analysis of serum N-glycans on a 96-well plate platform with dedicated database software. Anal. Biochem. 2008, 376, 1–12. 10.1016/j.ab.2007.12.012. 18194658

[ref29] Samal J.; Saldova R.; Rudd P. M.; Pandit A.; O’Flaherty R. Region-Specific Characterization of N-Glycans in the Striatum and Substantia Nigra of an Adult Rodent Brain. Anal. Chem. 2020, 92, 12842–12851. 10.1021/acs.analchem.0c01206. 32815717

[ref30] Küster B.; Wheeler S. F.; Hunter A. P.; Dwek R. A.; Harvey D. J. Sequencing of N-linked oligosaccharides directly from protein gels: in-gel deglycosylation followed by matrix-assisted laser desorption/ionization mass spectrometry and normal-phase high-performance liquid chromatography. Anal. Biochem. 1997, 250, 82–101. 10.1006/abio.1997.2199. 9234902

[ref31] Bigge J. C.; Patel T. P.; Bruce J. A.; Goulding P. N.; Charles S. M.; Parekh R. B. Nonselective and efficient fluorescent labeling of glycans using 2-amino benzamide and anthranilic acid. Anal. Biochem. 1995, 230, 229–238. 10.1006/abio.1995.1468. 7503412

[ref32] Adamczyk B.; Stöckmann H.; O’Flaherty R.; Karlsson N. G.; Rudd P. M. High-Throughput Analysis of the Plasma N-Glycome by UHPLC. Methods Mol. Biol. 2017, 1503, 97–108. 10.1007/978-1-4939-6493-2_8. 27743361

[ref33] Schulz B. L.; Packer N. H.; Karlsson N. G. Small-scale analysis of O-linked oligosaccharides from glycoproteins and mucins separated by gel electrophoresis. Anal. Chem. 2002, 74, 6088–6097. 10.1021/ac025890a. 12498206

[ref34] Kumagai T.; Katoh T.; Nix D. B.; Tiemeyer M.; Aoki K. In-Gel β-Elimination and Aqueous–Organic Partition for Improved O- and Sulfoglycomics. Anal. Chem. 2013, 85, 8692–8699. 10.1021/ac4015935. 23937624 PMC4004059

[ref35] Saldova R.; Asadi Shehni A.; Haakensen V. D.; Steinfeld I.; Hilliard M.; Kifer I.; Helland Å.; Yakhini Z.; Børresen-Dale A.-L.; Rudd P. M. Association of N-glycosylation with breast carcinoma and systemic features using high-resolution quantitative UPLC. J. Proteome Res. 2014, 13, 2314–2327. 10.1021/pr401092y. 24669823

[ref36] Tyanova S.; Temu T.; Sinitcyn P.; Carlson A.; Hein M. Y.; Geiger T.; Mann M.; Cox J. The Perseus computational platform for comprehensive analysis of (prote)omics data. Nat. Methods 2016, 13, 731–740. 10.1038/nmeth.3901. 27348712

[ref37] Endo T. Mammalian O-mannosyl glycans: Biochemistry and glycopathology. Proc. Jpn. Acad., Ser. B 2019, 95, 39–51. 10.2183/pjab.95.004. 30643095 PMC6395781

[ref38] Finne J. Structure of the O-glycosidically linked carbohydrate units of rat brain glycoproteins. Biochim. Biophys. Acta, Protein Struct. 1975, 412, 317–325. 10.1016/0005-2795(75)90046-x. 1191682

[ref39] Martin P. T. The dystroglycanopathies: the new disorders of O-linked glycosylation. Semin. Pediatr. Neurol. 2005, 12, 152–158. 10.1016/j.spen.2005.10.003. 16584074 PMC2860379

[ref40] Wang W.; Gopal S.; Pocock R.; Xiao Z. Glycan mimetics from natural products: new therapeutic opportunities for neurodegenerative disease. Molecules 2019, 24, 4604 10.3390/molecules24244604. PMC6943557 31888221

[ref41] Yaji S.; Manya H.; Nakagawa N.; Takematsu H.; Endo T.; Kannagi R.; Yoshihara T.; Asano M.; Oka S. Major glycan structure underlying expression of the Lewis X epitope in the developing brain is O-mannose-linked glycans on phosphacan/RPTPβ. Glycobiology 2015, 25, 376–385. 10.1093/glycob/cwu118. 25361541

[ref42] Kudo T.; Fujii T.; Ikegami S.; Inokuchi K.; Takayama Y.; Ikehara Y.; Nishihara S.; Togayachi A.; Takahashi S.; Tachibana K.; Yuasa S.; Narimatsu H. Mice lacking α1,3-fucosyltransferase IX demonstrate disappearance of Lewis x structure in brain and increased anxiety-like behaviors. Glycobiology 2007, 17, 1–9. 10.1093/glycob/cwl047. 16973732

[ref43] Chou D. K. H.; Prasadarao N.; Koul O.; Jungalwala F. B. Developmental expression of HNK-1-reactive antigens in rat cerebral cortex and molecular heterogeneity of sulfoglucuronylneolactotetraosylceramide in CNS versus PNS. J. Neurochem. 1991, 57, 852–859. 10.1111/j.1471-4159.1991.tb08229.x. 1713615

[ref44] Terayama K.; Oka S.; Seiki T.; Miki Y.; Nakamura A.; Kozutsumi Y.; Takio K.; Kawasaki T. Cloning and functional expression of a novel glucuronyltransferase involved in the biosynthesis of the carbohydrate epitope HNK-1. Proc. Natl. Acad. Sci. U.S.A. 1997, 94, 6093 10.1073/pnas.94.12.6093. 9177175 PMC21007

[ref45] Cagnoni A. J.; Pérez Sáez J. M.; Rabinovich G. A.; Mariño K. V. Turning-off signaling by siglecs, selectins, and galectins: chemical inhibition of glycan-dependent interactions in cancer. Front. Oncol. 2016, 6, 109 10.3389/fonc.2016.00109. 27242953 PMC4865499

[ref46] Brockhausen I. Pathways of O-glycan biosynthesis in cancer cells. Biochim. Biophys. Acta 1999, 1473, 67–95. 10.1016/s0304-4165(99)00170-1. 10580130

[ref47] Limón I. D.; Ramírez E.; Díaz A.; Mendieta L.; Mayoral M. Á.; Espinosa B.; Guevara J.; Zenteno E. Alteration of the sialylation pattern and memory deficits by injection of Aβ(25-35) into the hippocampus of rats. Neurosci. Lett. 2011, 495, 11–16. 10.1016/j.neulet.2011.03.006. 21419829

[ref48] Yoshimura T.; Hayashi A.; Handa-Narumi M.; Yagi H.; Ohno N.; Koike T.; Yamaguchi Y.; Uchimura K.; Kadomatsu K.; Sedzik J.; Kitamura K.; Kato K.; Trapp B. D.; Baba H.; Ikenaka K. GlcNAc6ST-1 regulates sulfation of N-glycans and myelination in the peripheral nervous system. Sci. Rep. 2017, 7, 42257 10.1038/srep42257. 28186137 PMC5301494

[ref49] Hoban D. B.; Newland B.; Moloney T. C.; Howard L.; Pandit A.; Dowd E. The reduction in immunogenicity of neurotrophin overexpressing stem cells after intra-striatal transplantation by encapsulation in an in situ gelling collagen hydrogel. Biomaterials 2013, 34, 9420–9429. 10.1016/j.biomaterials.2013.08.073. 24054846

[ref50] Moriarty N.; Pandit A.; Dowd E. Encapsulation of primary dopaminergic neurons in a GDNF-loaded collagen hydrogel increases their survival, re-innervation and function after intra-striatal transplantation. Sci. Rep. 2017, 7, 16033 10.1038/s41598-017-15970-w. 29167483 PMC5700093

[ref51] van Halbeek H.; Strang A.-M.; Lhermitte M.; Rahmoune H.; Lamblin G.; Roussel P. Structures of monosialyl oligosaccharides isolated from the respiratory mucins of a non-secretor (O, Lea+b-) patient suffering from chronic bronchitis. Characterization of a novel type of mucin carbohydrate core structure. Glycobiology 1994, 4, 203–209. 10.1093/glycob/4.2.203. 8054719

[ref52] Galili U. The alpha-gal epitope and the anti-Gal antibody in xenotransplantation and in cancer immunotherapy. Immunol. Cell Biol. 2005, 83, 674–686. 10.1111/j.1440-1711.2005.01366.x. 16266320

[ref53] St-Amour I.; Paré I.; Alata W.; Coulombe K.; Ringuette-Goulet C.; Drouin-Ouellet J.; Vandal M.; Soulet D.; Bazin R.; Calon F. Brain bioavailability of human intravenous immunoglobulin and its transport through the murine blood-brain barrier. J. Cereb. Blood Flow Metab. 2013, 33, 1983–1992. 10.1038/jcbfm.2013.160. 24045402 PMC3851908

[ref54] Watanabe Y.; Aoki-Kinoshita K. F.; Ishihama Y.; Okuda S. GlycoPOST realizes FAIR principles for glycomics mass spectrometry data. Nucleic Acids Res. 2021, 49, D1523–D1528. 10.1093/nar/gkaa1012. 33174597 PMC7778884

