# Supplementary material for: The O-Glycome of Human Nigrostriatal Tissue and Its Alteration in Parkinson’s Disease
Source: J Proteome Res. 2021 Jun 30;20(8):3913–24. doi: 10.1021/acs.jproteome.1c00219 (PMC8353623; doi:10.1021/acs.jproteome.1c00219)
Supplement: Supplementary file 1 — pr1c00219_si_001.pdf [file pr1c00219_si_001.pdf]

SUPPORTING INFORMATION: The *O*-Glycome of Human Nigrostriatal Tissue and its Alteration in Parkinson's Disease

Hayden Wilkinson[1,2,3], Kristina A. Thomsson[4], Ana L. Rebelo[2], Mark Hilliard[1], Abhay Pandit[2], Pauline M. Rudd[1], Niclas G. Karlsson[4,5], *Radka Saldova[1,2,3]

[1]NIBRT GlycoScience Group, National Institute for Bioprocessing, Research and Training, Blackrock, Dublin A94 X099, Ireland

[2]CÚRAM, SFI Research Centre for Medical Devices, National University of Ireland, Galway H91 W2TY, Ireland

[3]UCD School of Medicine, College of Health and Agricultural Science, University College Dublin, Dublin D07 A8NN, Ireland

[4]Department of Medical Biochemistry and Cell Biology, Institute of Biomedicine, Sahlgrenska Academy, University of Gothenburg, Gothenburg 405 30, Sweden

[5]Department of Life Sciences and Health, Faculty of Health Sciences, Oslo Metropolitan University, Oslo 0167, Norway

***Corresponding author:** Dr Radka Fahey (Saldova), NIBRT GlycoScience Group, National Institute for Bioprocessing, Research and Training, Blackrock, Dublin A94 X099, Ireland, radka.fahey@nibrt.ie, phone: +353 1 215 8147.

Supplementary Discussion…………………………………………………………. S-3 – S-5

Supplementary Figures……………………………………………………………….. S-6 – S-20

Supplementary Tables………………………………………………………………... S-21 – S-27

Supplementary Information References…………………..……………………... S-28 – S-29

Supplementary Spreadsheet………………………………………………………. Additional File

Supplementary Discussion – additional discussion outlining analytical considerations for characterization as well as potential improvements for the non-reductive *O*-glycan release method.

Supplementary Figures – additional figures mentioned, but not included, in the manuscript body.

Supplementary Tables – additional tables mentioned, but not included, in the manuscript body.

Supplementary Spreadsheet – larger datasets that were unsuitable for condensing into the "Supplementary Tables" section, namely, Supplementary Tables 5 – 9.

SUPPLEMENTARY DISCUSSION

Characterization Considerations

Studies have historically focused on *O*-GlcNAcylation rather than complete *O*-glycosylation of PD associated tissue, however, the results presented in this work outline the complexity of the *O*-glycome of striatum and substantia nigra as well as significant glycosylation changes occurring after the onset of PD. While 70 glycan structures were identified, considerations were made. For instance, as observed in Supplementary Table 2, characterization of some glycan structures was made using only a single characterization method (exoglycosidase digestion *or* QToF experiments *or* ion-trap experiments) and lacked confirmation from complementary methods. The commonly applied analytical method used most frequently in the literature to identify released *O*-glycans (MS^1 and MS^2 analysis of glycan alditols from reductive release) confidently confirmed 21 glycan structures, with the remainder derived from exoglycosidase digestions and/or MS^1 confirmation of similarly separated (HILIC) 2AB labelled structures (analyzed with QToF-MS). Reasons for lack of orthogonality were due in part to sample concentration limitations, limiting what could be detected on some systems, but may also be due to differences in sensitivity of detection particularly for the identification of 2AB labelled glycans in MS using the Xevo QToF MS. In negative ion mode, detection of negatively charged glycans (those with sulfation and sialylation) provided greater sensitivity compared with neutral glycans[1]. Furthermore, the available glycan sample was so low that a total ion chromatogram of the glycans (from QToF experiment on 2AB labelled glycans) could not be resolved from the baseline (requiring mining of extracted ion chromatograms), ultimately limiting what could be detected in MS. In future work, orthogonal methods of characterization for instance CE, NMR and further MS^2 analysis of HILIC-separated 2AB labeled *O*-glycans at similar elution gradients and at greater concentrations may be used to help validate results. Additionally, if persisting with non-reductive *O*-glycan release methods, incorporating a more sensitive glycan label (like Procainamide[2] or RapiFlour-MS[3]) may help resolve glycan structures lost in the baseline of UPLC chromatograms and MS spectra. This, coupled with the apparent need to limit peeling through further optimization, may aid in characterization.

Exoglycosidase Limitation

During data interpretation, there was growing evidence to suggest some enzymes were poorly digesting some glycan structures or simply not digesting them at all. It was determined that BTG (β1-3,4 galactosidase) had very low activity on cleaving β1-3 linked galactose if it was linked directly to a core GalNAc (the mucin-type core 1 disaccharide). This did not seem to affect other disaccharides that contained different linkages or were composed of different monosaccharides, such as GlcNAcβ1-6GalNAc or GlcNAcβ1-2man. Furthermore, there was severe inhibition of all galactosidases on β-linked galactose if the galactose was linked directly to a GlcNAc that itself was linked to a fucose. Galactose, in this instance, would only digest once the internal fucose was removed using AMF (α1-3,4 fucosidase) or BKF (α1-2,3,4,6 fucosidase). This appeared not to be the case for ga3[ga4(fa3GL6)]**GA** however, which de-galactosylated without initial fucosidase treatment. Also present in some samples was a lactose (Galβ1-4Glc) contaminating peak which can be seen in some chromatograms at GU 1.96. In this case, SPG which targets specifically β1-4 linked galactose had little to no enzymatic activity, however BTG with its broader specificity for β1-4 and β1-3 could cleave the structure (this is why 2AB-glucose is present in the glycan list). Royle et al., 2002 has previously reported some of these observations[4] for *O*-glycans and Guile et al., 1998[5] for *N*-glycans. It is suggested that, for the case of the non-digestible disaccharides, the process of reductively aminating with 2AB forces the core monosaccharide into its open-ring conformation, which may inhibit recognition by specific exoglycosidases. In future studies, experiments with labelling reagents that leave the core monosaccharide in its closed-ring cyclic form (such as AQC or RapiFluor-MS) will need to be performed and compared with this method.

Microwave-assisted Non-Reductive *O*-Glycan Release

While results were comparable to similar ammonia-based non-reductive β-elimination techniques seen in the literature[6-8], peeling from these methods remained a pressing issue. Through optimization, peeling was reduced to levels expected from a typical hydrazine based non-reductive release (approx. 15-20%), which fell more in line with non-reductively released *O*-glycans commercially available (Ludger™). Moving forward to clinical samples in (PD/ILBD/healthy control), the major release and analysis

methods were performed using this optimized ammonia-based non reductive release with 2AB labelling, however, reductive elimination was also performed for ion-trap MS experiments, which was advantageous as peeling here was negligible (no fluorescence detection however).

Due to the high number of variables that were tested in this work, many substitutions could be made to limit peeling from non-reductive β-elimination even further (whether it be identifying a more suitable alkaline reaction solution, or incorporating a more rapid tagging reagent for example). Another method to explore in the future is the creation of a flow system that removes *O*-glycans from the alkaline reaction solution immediately after release to limit peeling, as has been observed in the literature[9]. Furthermore, low yields remained an issue, particularly during exoglycosidase digestion studies where larger sample sizes were required. In future work, release efficiency studies will need to be performed and the method further optimized.

Additionally, glycoproteomic approaches to glycan analysis by leaving the glycan attached to the peptide glycosylation site have proven valuable in characterizing not only the glycans but also their relative position as they appear on the native protein[10]. Similarly, as glycoproteomic databases are built out, predictive technologies are also being employed to help make obsolete the non-reductive and reductive release systems.

SUPPLEMENTARY FIGURES

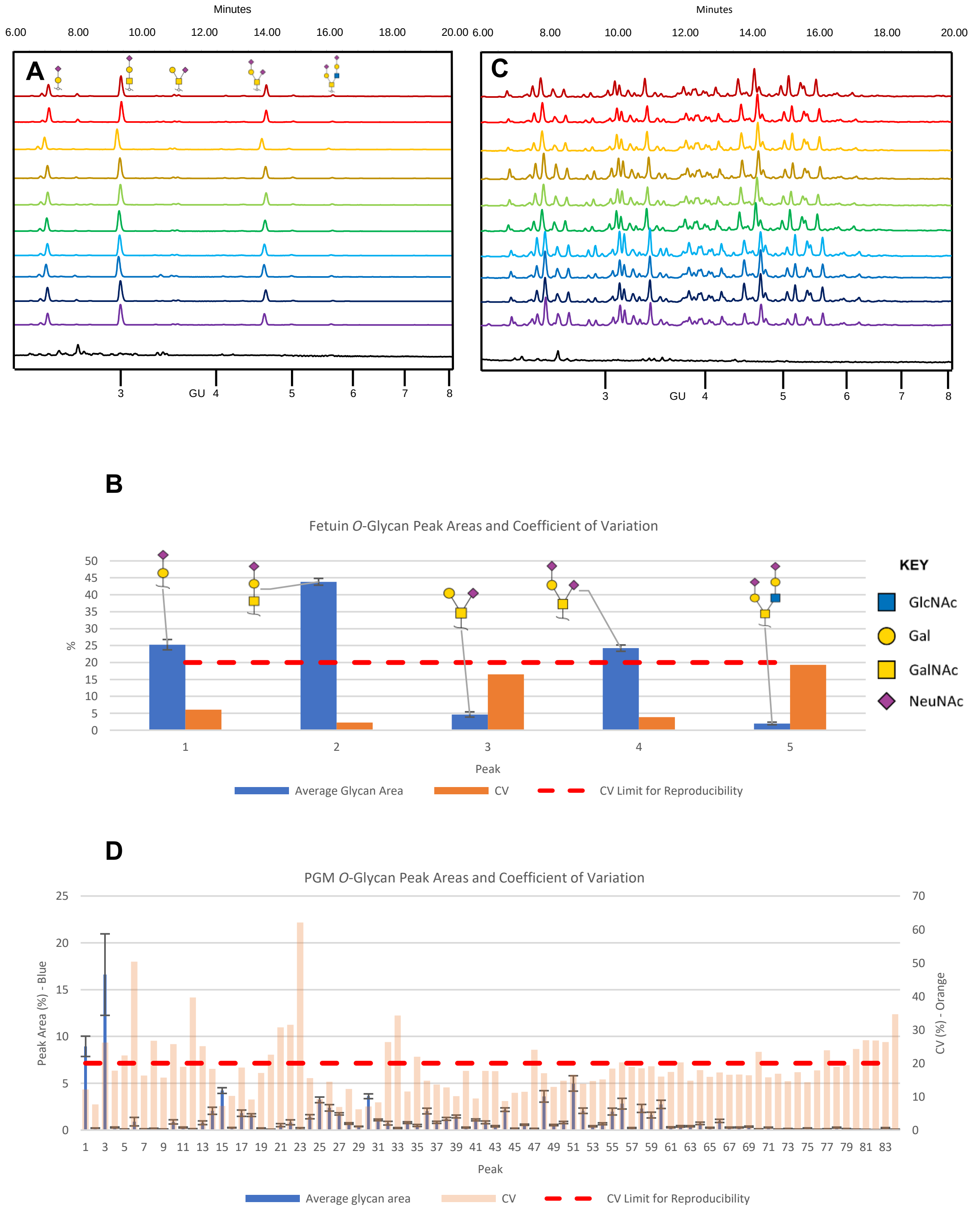

Supplementary Figure 1. *Development of a Microwave-Assisted Non-Reductive β-Elimination O-Glycan Release. (A) 10x replicate chromatograms of 2AB-labeled O-glycans from fetuin (blank in black) analyzed with FLR-HILIC-UPLC. (B) relative % area and CV for fetuin O-glycan replicates, (C) 10x replicate chromatograms from O-glycans from porcine gastric mucin (PGM) (blank in black) and (D) relative % area and CV for PGM replicates. Dotted line represents 20% CV limit (referenced against rightmost y axis). Symbol nomenclature is according to SNFG.*

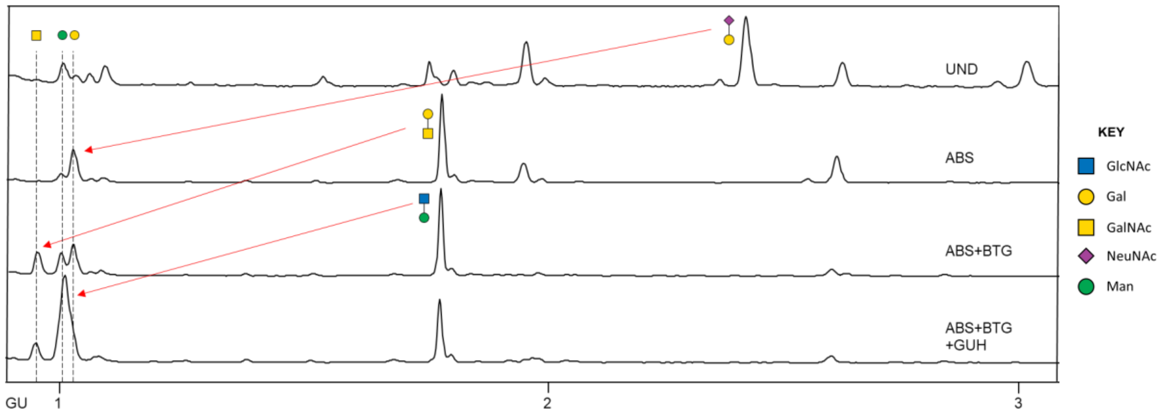

Supplementary Figure 2. *Monosaccharide elution order from exoglycosidase digestions of O-glycans from PD substantia nigra, released with nonreductive release and analyzed as 2AB-labeled derivatives with FLR-HILIC-UPLC. Partial digestion of disaccharides (primarily the T-antigen) at GU 1.8 remains throughout. UND – undigested native profile. Glycosidase abbreviations: ABS – α2-3,6,8,9 sialidase, BTG – β1-3,4 galactosidase, GUH – β1-2,3,4,6 N-acetylglucosaminidase. Symbol nomenclature is according to SNFG.*

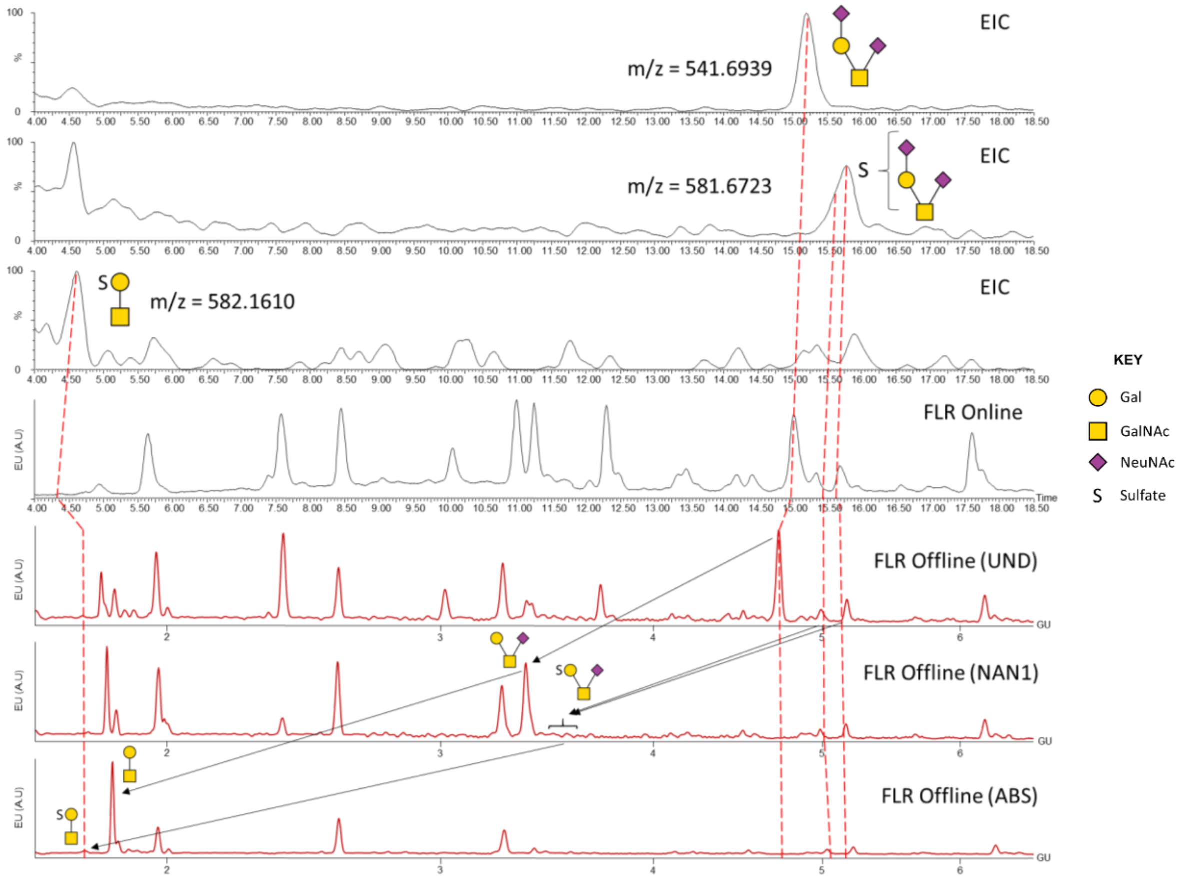

Supplementary Figure 3. *Offline exoglycoasidase chromatograms of nonreductively released 2AB-labeled O-glycans from PD substantia nigra pool alongside extracted ion chromatograms (EIC) and online FLR chromatogram of FLR-HILIC-UPLC-ESI-MS analysis. Exoglycosidase chromatograms shown are UND-undigested, NAN1-α2-3 sialidase digested and ABS- α2-3,6,8,9 sialidase digested. Disialyl T-antigen (m/z 541 – doubly charged) shown across chromatograms with suspected sulfated analogues (m/z 581 – doubly charged; two proposed structures here as indicated by the EIC peak with observable fronting/asymmetry). Non sulfated sialyl T-antigen is predominant product of the FLR-HILIC-UPLC peak as indicated by complete digestion with NAN1, and sulfated analogues suggested to be smaller components of their associated peaks (as shown by incomplete peak digestion with NAN1). Further ABS digestion identifies peak shift of sulfated and non-sulfated trisaccharides after removal of the α2-6 linked sialic acid – shifting to their respective positions. Confirmation of the non-sulfated disaccharide was achieved through further galactosidase digestion and confirmation of the sulfated*

disaccharide was proposed through comparison with EIC of the same structure (m/z 582 – singly charged) in FLR-HILIC-UPLC-ESI-MS analysis. Symbol nomenclature is according to SNFG.

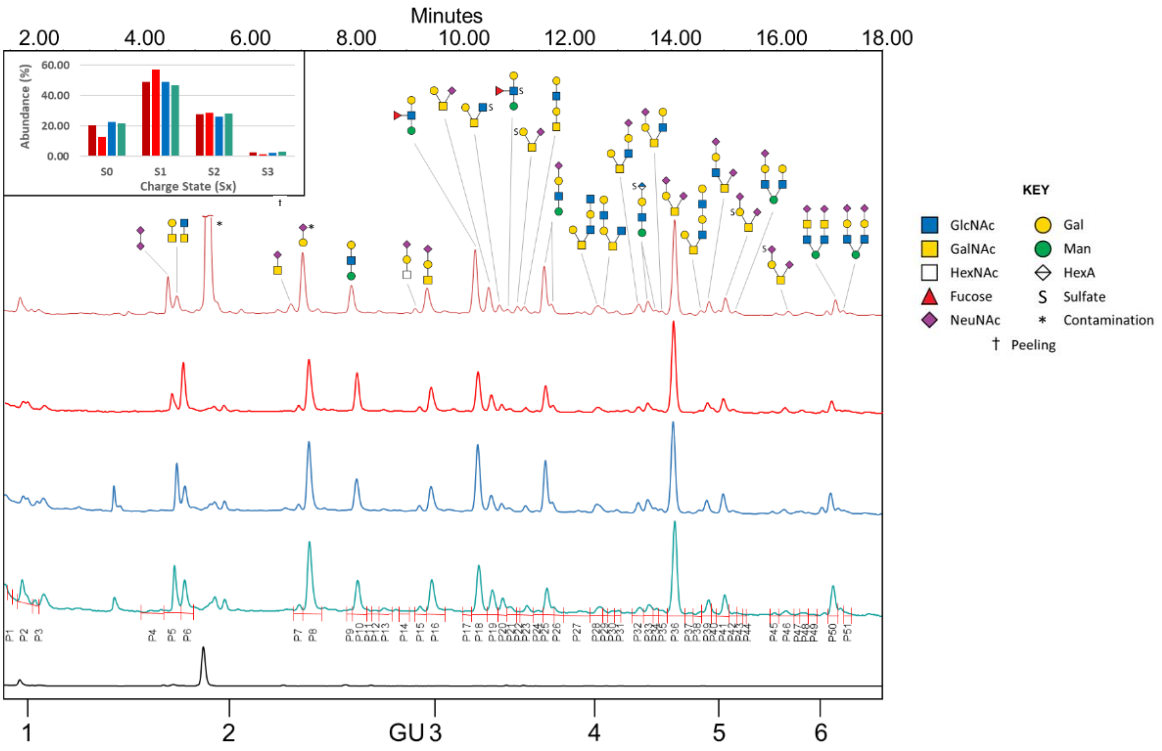

Supplementary Figure 4. *Representative FLR-HILIC-UPLC chromatograms of 2AB-labeled O-glycans released using nonreductive release from human striatum (healthy – dark red, PD – light red) and substantia nigra (healthy – dark blue, PD – green). Blank - black. Integrated peaks on chromatograms shown in PD substantia nigra – can be referenced against Supplementary Table 3. Inset FLR-WAX-UPLC graph shows relative abundance of 2AB labeled glycans by charge state (Sx) (bar colours correspond to chromatogram colours). FLR-WAX-UPLC chromatograms shown in Supplementary Figure 4. Symbol nomenclature is according to SNFG.*

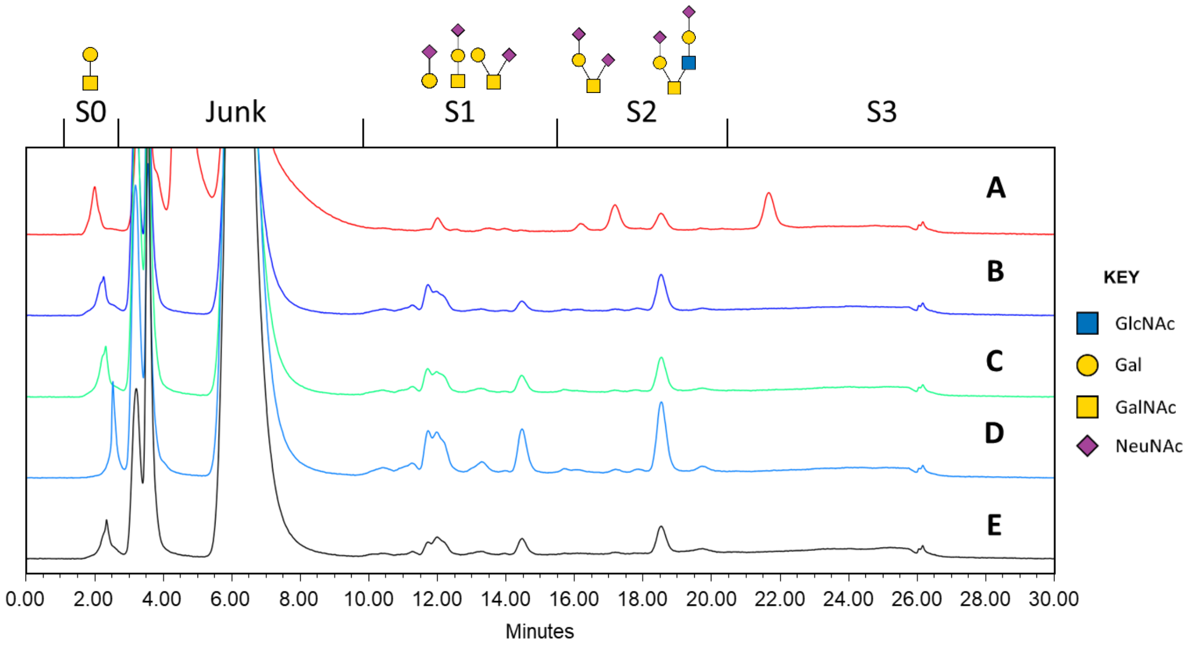

Supplementary Figure 5. *FLR-WAX-UPLC Chromatograms of nonreductively released 2AB-labeled O-glycans from (A) Fetuin, (B) healthy striatum, (C) PD striatum, (D) healthy substantia nigra, (E) PD substantia nigra. S0, S1, S2 and S3 indicates the elution times for glycans with 1, 2 or 3 charged residues (sulfates or NeuNAc), respectively, and has been validated using fetuin glycan standards. SNFG glycans represented are O-glycans expected from fetuin.*

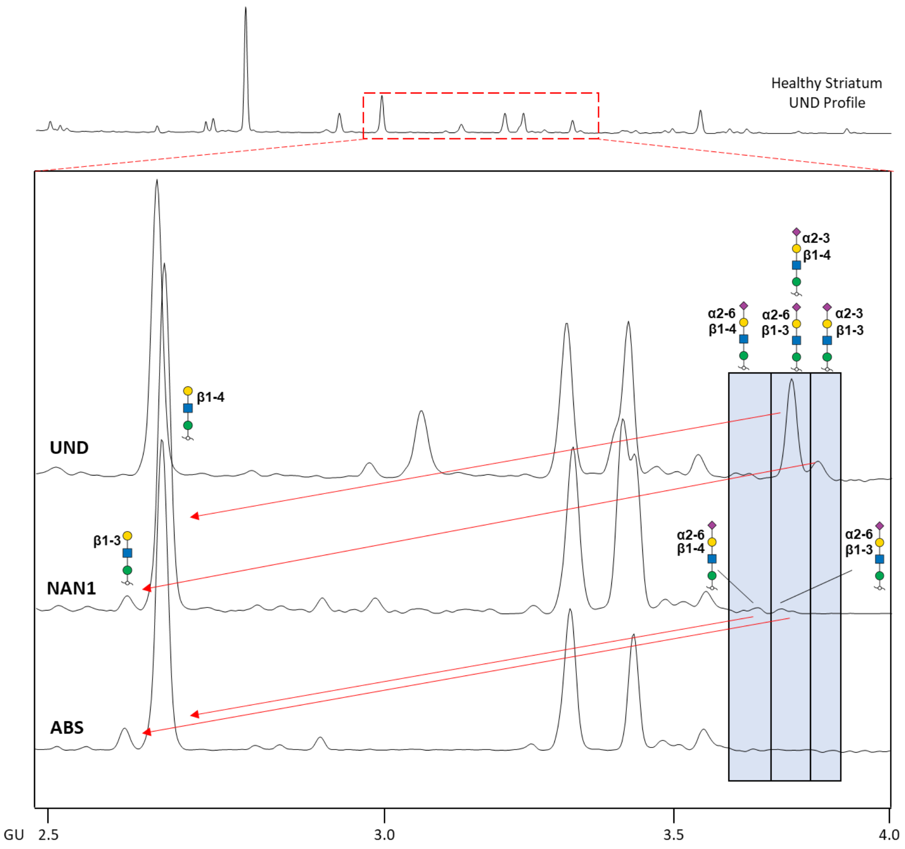

Supplementary Figure 6. *Digestion profile from FLR-HILIC-UPLC of sialylated mannose core tetrasaccharide isomers from the healthy striatum. Linkage information of each sialic acid and galactose residue shown. UND – undigested native profile. NAN1 – α2-3 sialidase treated profile. ABS – α2-3,6,8,9 sialidase treated profile. Symbol nomenclature is according to SNFG.*

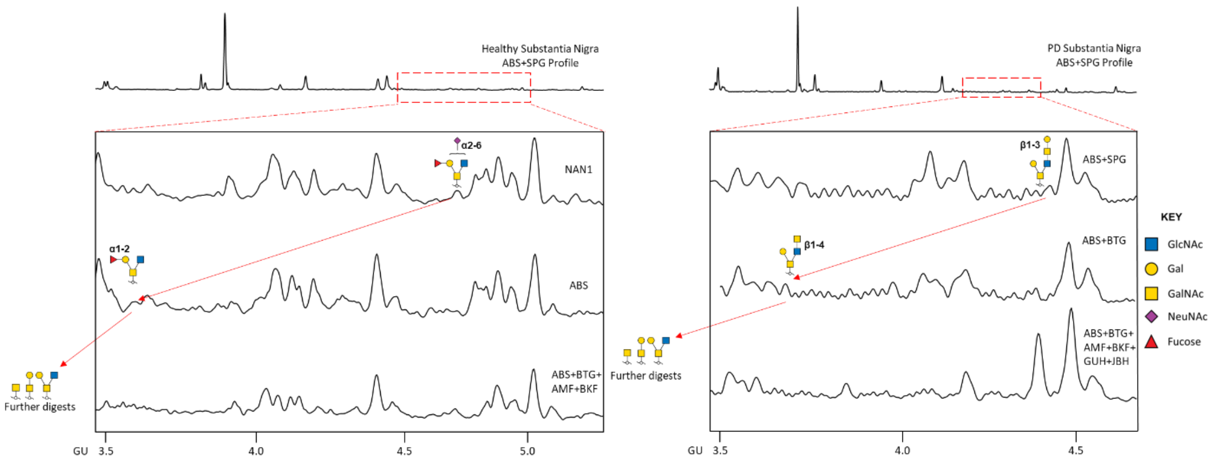

Supplementary Figure 7. *Glycosidase digestion profiles of 2AB-labeled O-glycans interpreted as NeuNAcα2-6-(Fucα1-2Galβ1-3)[GlcNAcβ1-6]GalNAc (left) and Galβ1-3[Galβ1-3GalNAcβ1-4GlcNAcβ1-6]GalNAc (right) from Healthy and PD substantia nigra O-glycan pools, respectively . The O-glycans were released with nonreductive release and analyzed as 2AB-labeled derivatives with FLR-HILIC-UPLC. Glycosidase abbreviations: NAN1 – α2-3 sialidase, ABS – α2-3,6,8,9 sialidase, SPG – β1-4 galactosidase, BTG – β1-3,4 galactosidase, AMF – α1-3,4 fucosidase, BKF – α1-2,3,4,6 fucosidase, GUH – β1-2,3,4,6 N-acetylglucosaminidase, JBH – β1-3,4,6-N-acetylhexosaminidase. Symbol nomenclature is according to SNFG.*

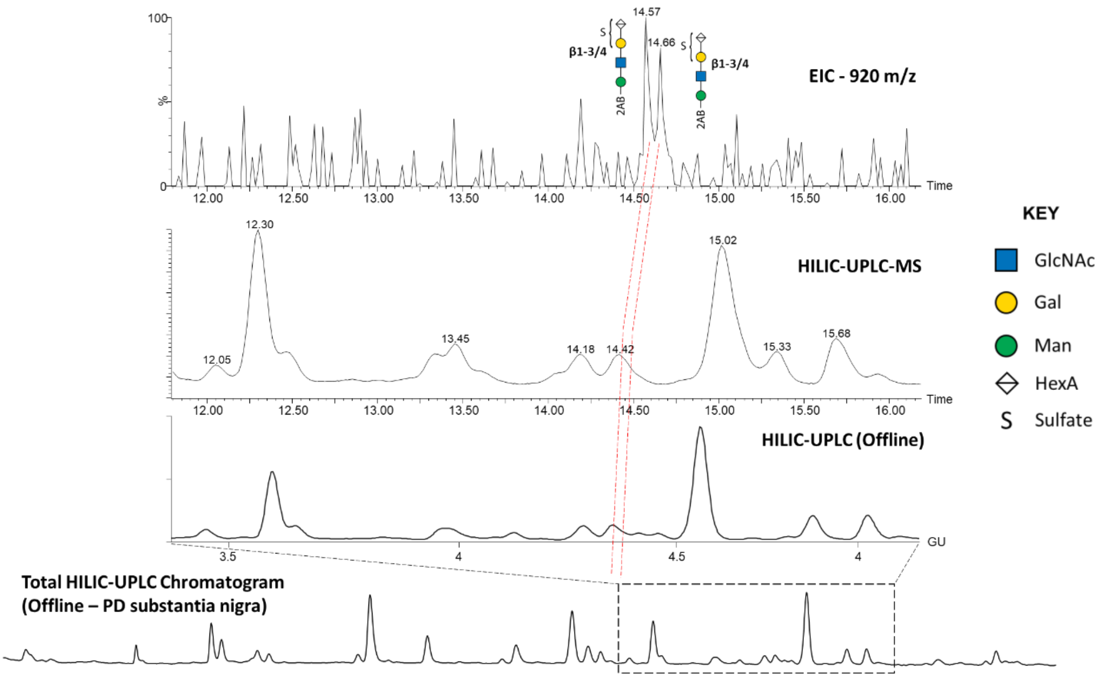

Supplementary Figure 8. *Comparing 2AB-labeled sulfated glycans (HexA-Gal-GlcNAc-Man) from FLR-HILIC-UPLC-MS spectra to offline FLR-HILIC-UPLC profiles. Confirmed by consistent non-digestible peak area at GU 4.4 in exoglycosidase panels (supplementary figures 3-10). Delay of approximately 0.15 minutes between FLR detection and MS detection. Symbol nomenclature is according to SNFG.*

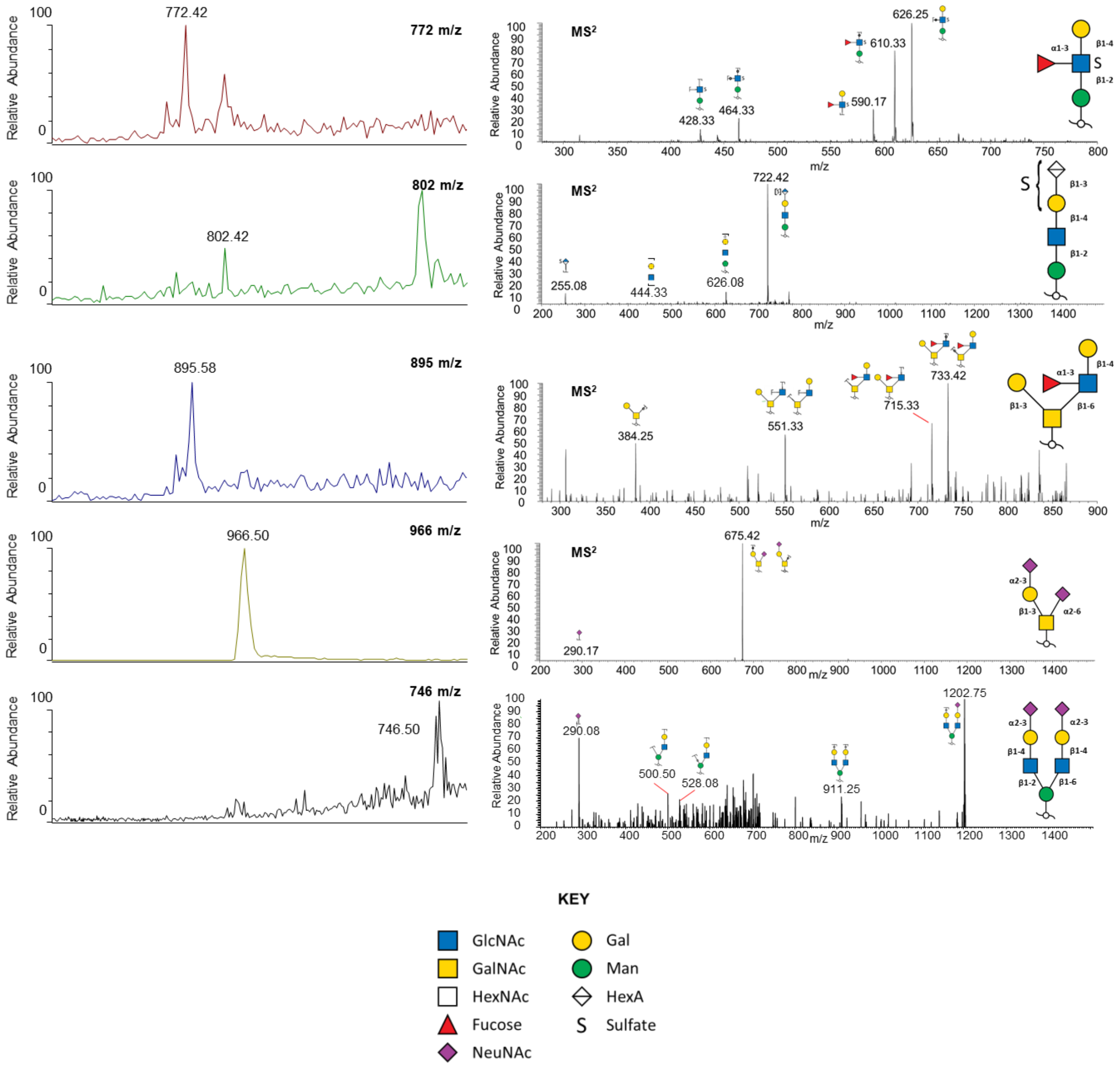

Supplementary Figure 9. *Extracted ion chromatograms and MS^2 spectra of selected O-glycans released from healthy striatum tissue using reductive release. The O-glycans were detected as monocharged or dicharged precursor ions ([M-H]$^-$ and [M-2H]$^{2-}$). They were interpreted as Galβ1-4(Fucα1-3-HSO3-GlcNAcβ1-2Manol (772 m/z, [M-H]$^-$), HSO3-HexAβ1-3-Gal β1-4GlcNAcβ1-2Manol – resembling the sulfated Human Natural Killer-1 epitope-containing mannose core tetrasaccharide (802 m/z [M-H]$^-$), Galβ1-3[Galβ1-4(Fucα1-3GlcNAcβ1-6)]GalNAcol (895 m/z, [M-H]$^-$), NeuNAcα2-3Galβ1-3[NeuNAcα2-6]GalNAcol (966 m/z, [M-H]$^-$; most common glycan in each sample) and NeuNAcα2-3Galβ1-4GlcNAcβ1-2[NeuNAcα2-3Galβ1-4GlcNAcβ1-6]Manol (746 m/z, [M-2H]$^{2-}$; from PD substantia nigra). Linkage determined using a combination of exoglycosidase treatment of non-*

reductively released 2AB-labeled glycans as well as MS^3 analysis of reductively released glycan alditols. Symbol nomenclature is according to SNFG.

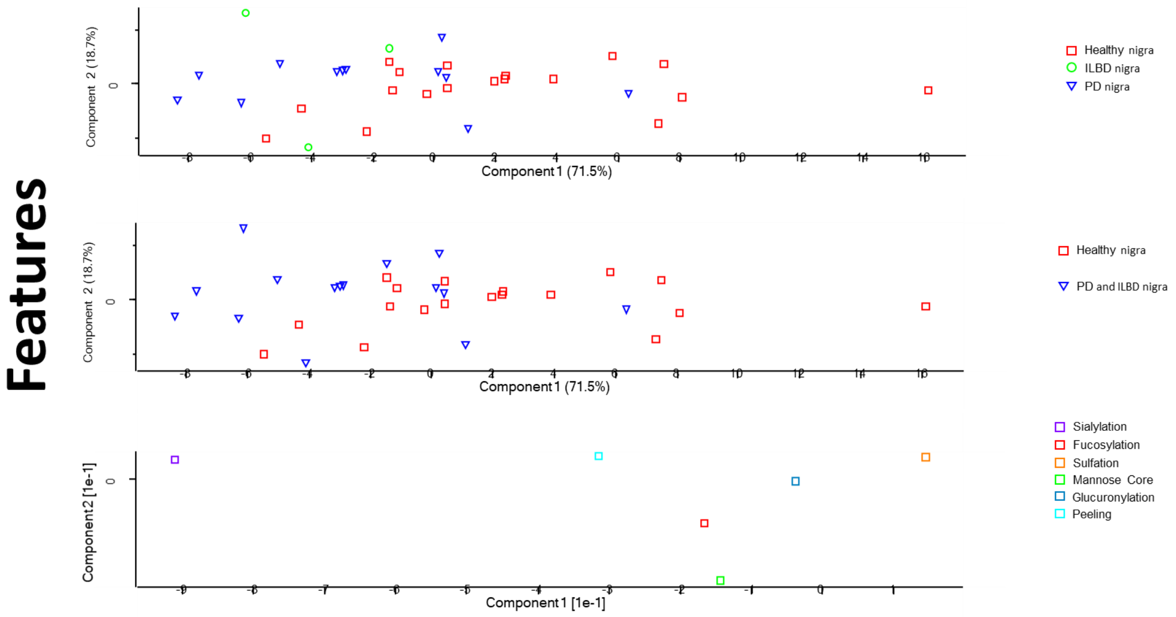

Supplementary Figure 10. *PCA comparing glycan features of individual samples in healthy control, PD and ILDB substantia nigra tissues. Upper plot shows separation of each group relative to the features in the lower plot. Middle plot omits ILBD samples.*

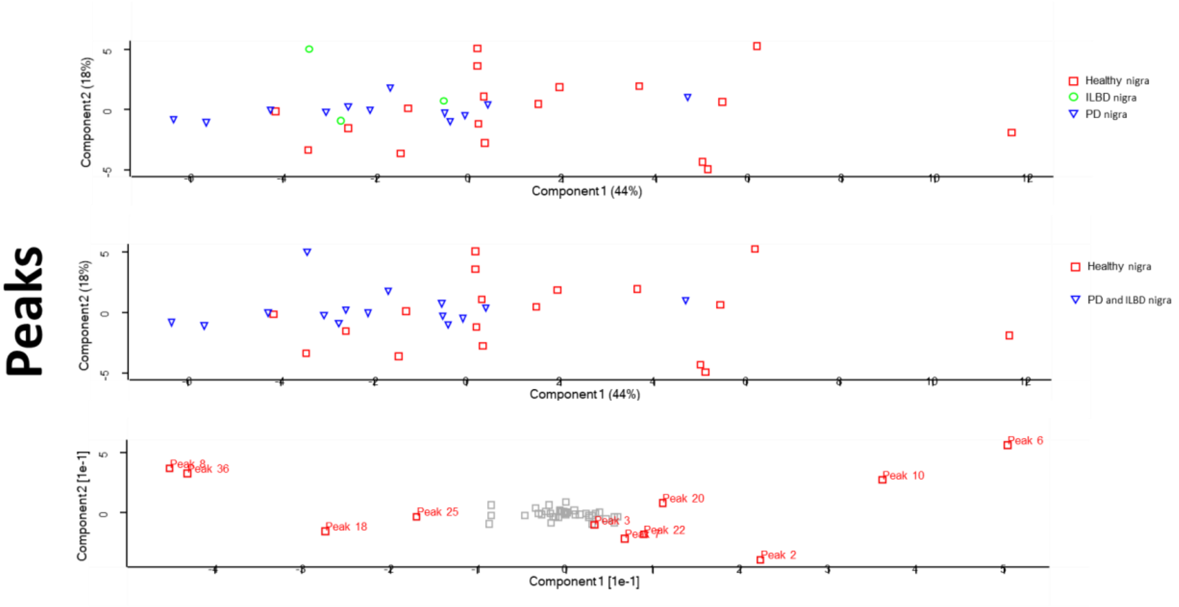

Supplementary Figure 11. *PCA comparing glycan peaks of individual samples in healthy control, PD and ILDB substantia nigra tissues. Upper plot shows separation of each group relative to the peaks in the lower plot. Middle plot omits ILBD samples.*

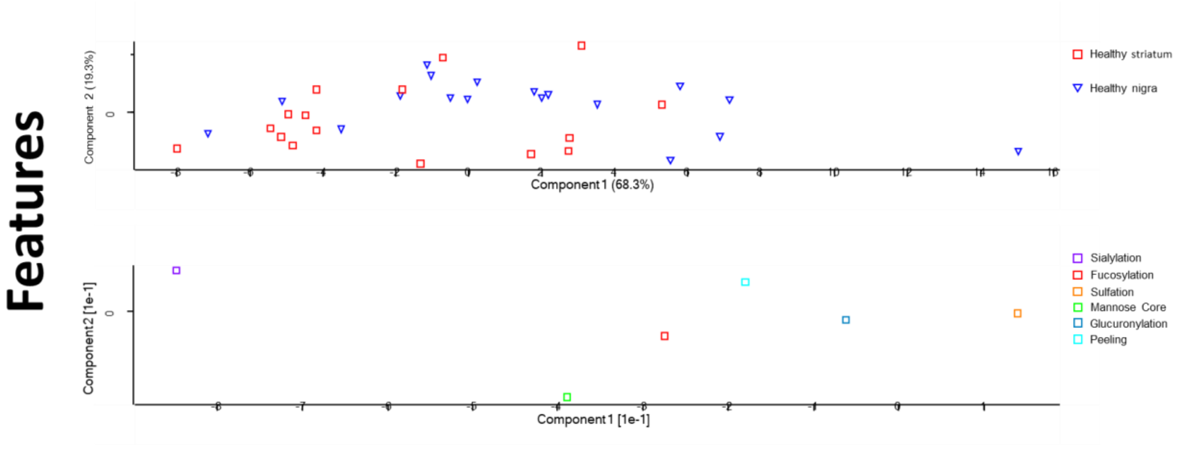

Supplementary Figure 12. *PCA comparing glycan features between individual samples in healthy striatum and substantia nigra tissues. Upper plot shows separation of each group relative to the features in the lower plot.*

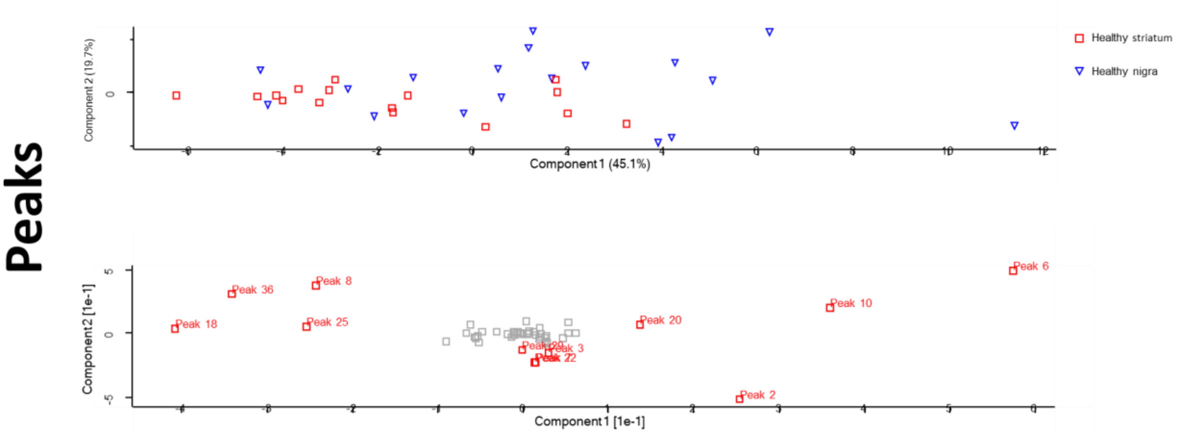

Supplementary Figure 13. *PCA comparing glycan peaks between individual samples in healthy striatum and substantia nigra tissues. Upper plot shows separation of each group relative to the peaks in the lower plot.*

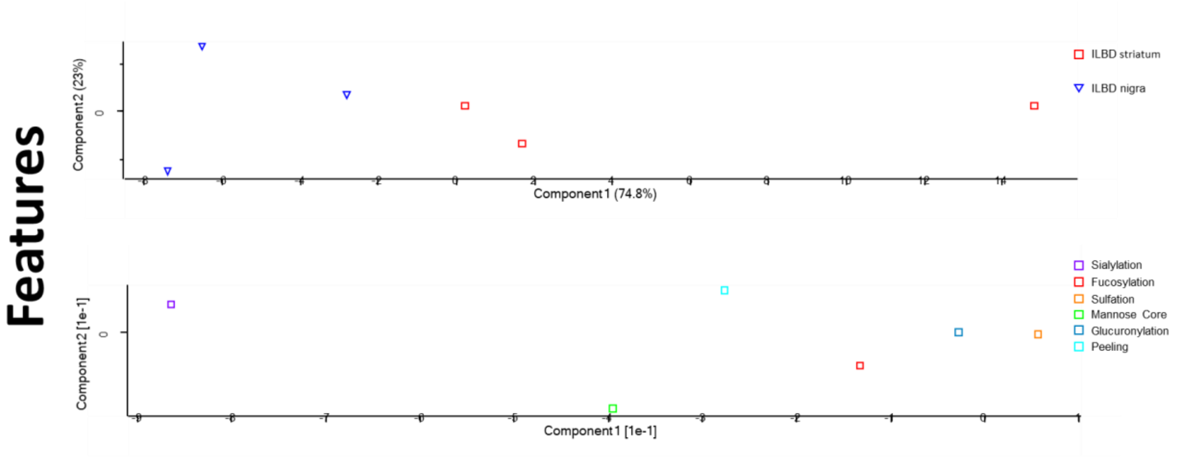

Supplementary Figure 14. *PCA comparing glycan features between individual samples in ILBD striatum and substantia nigra tissues. Upper plot shows separation of each group relative to the features in the lower plot.*

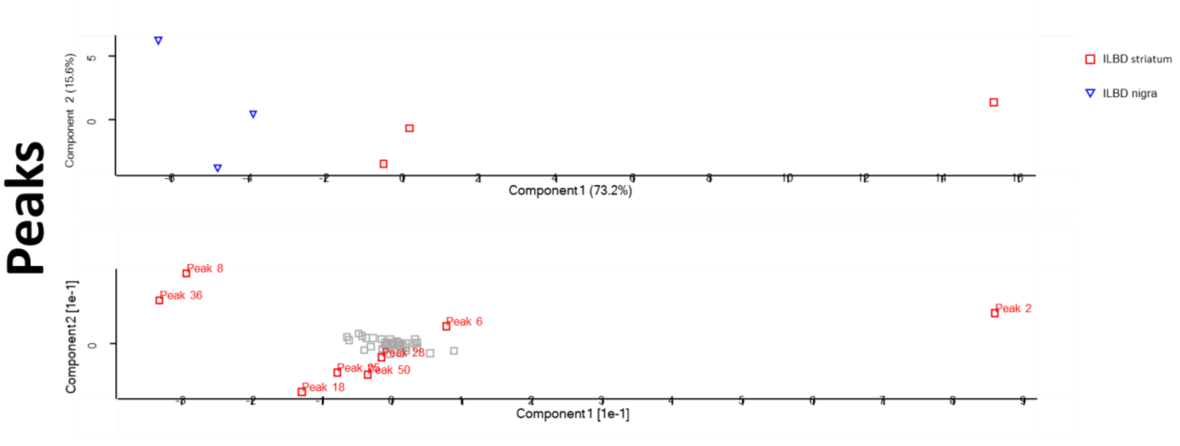

Supplementary Figure 15. *PCA comparing glycan peaks between individual samples in ILBD striatum and substantia nigra tissues. Upper plot shows separation of each group relative to the peaks in the lower plot.*

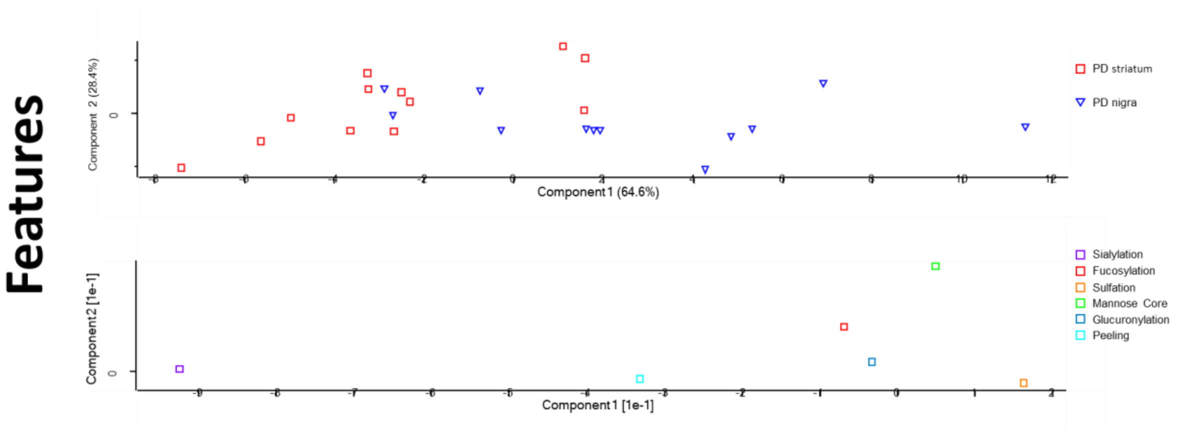

Supplementary Figure 16. *PCA comparing glycan features between individual samples in PD striatum and substantia nigra tissues. Upper plot shows separation of each group relative to the features in the lower plot.*

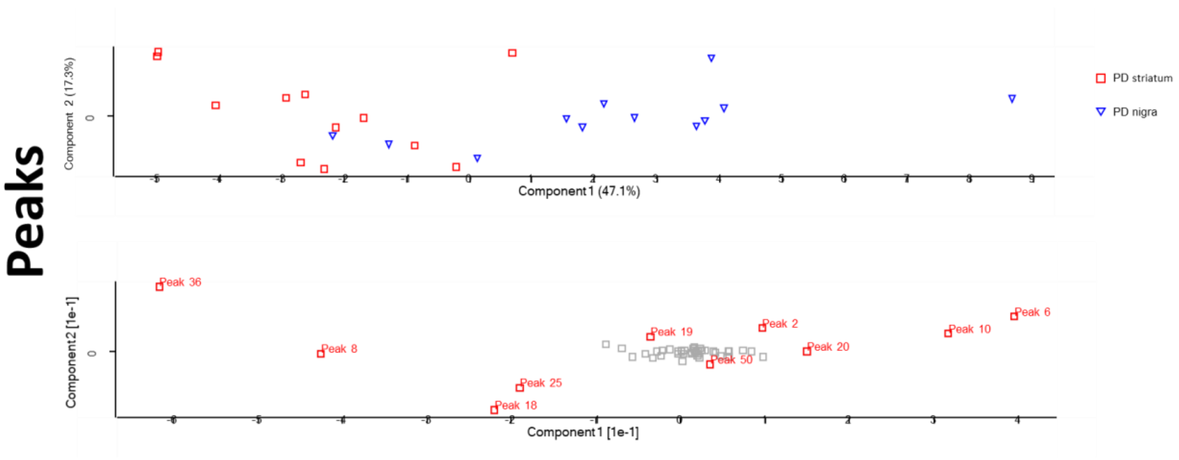

Supplementary Figure 17. *PCA comparing glycan peaks between individual samples in PD striatum and substantia nigra tissues. Upper plot shows separation of each group relative to the peaks in the lower plot.*

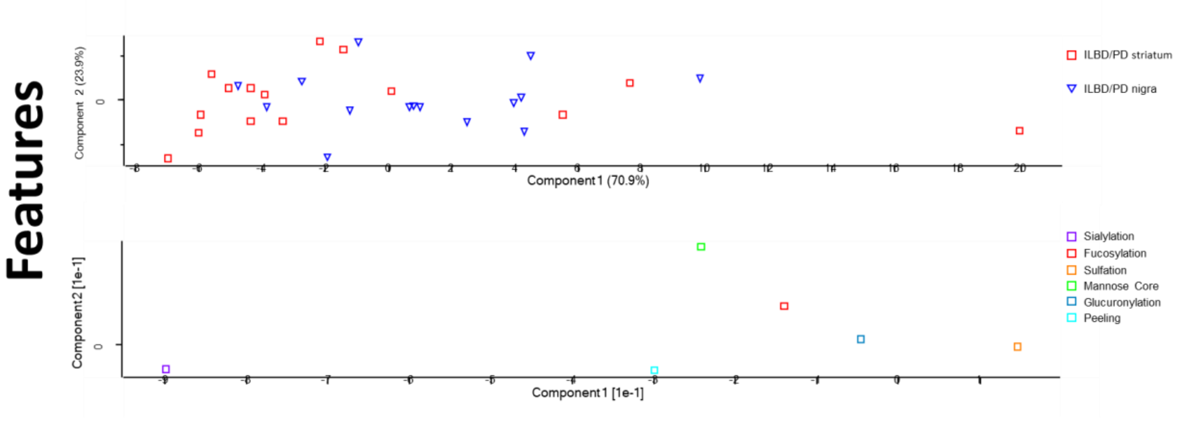

Supplementary Figure 18. *PCA comparing glycan features between individual samples in ILBD and PD (pooled together) striatum and substantia nigra tissues. Upper plot shows separation of each group relative to the features in the lower plot.*

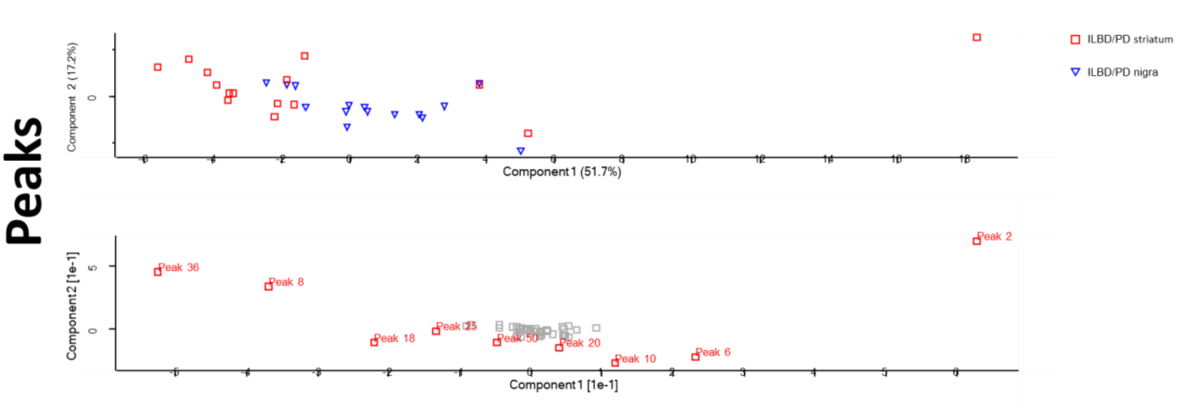

Supplementary Figure 19. *PCA comparing glycan peaks between individual samples in ILBD and PD (pooled together) striatum and substantia nigra tissues. Upper plot shows separation of each group relative to the peaks in the lower plot.*

SUPPLEMENTARY TABLES

Brain Bank identifier number	Gender	Age at death (years)	Braak stage	Group
PD204	F	86	3	PD
PD572	M	72	3	
PD576	F	80	3	
PD579	M	76	4	
PD590	F	82	3	
PD591	M	77	4	
PD596	M	85	4	
PD612	M	78	4	
PD683	F	83	4	
PD687	M	86	3	
PD709	F	88	3	
PD712	F	87	4	
PDC007	F	104	ILBD	Incidental Lewy Bodies Disease (ILBD)
PDC082	F	77	ILBD	
PDC108	F	82	ILBD	
PDC034	M	90	N/A	Healthy
PDC040	F	61	N/A	
PDC053	F	89	N/A	
PDC085	F	82	N/A	
PDC088	F	96	N/A	
PDC105	M	95	N/A	
PDC107	F	87	N/A	
PDC111	F	88	N/A	
PD291A	F	87	N/A	
C045	M	77	N/A	
C054	M	66	N/A	
C064	F	63	N/A	
C074	F	84	N/A	
C075	M	88	N/A	
C083	F	87	N/A	
C084	F	84	N/A	
C085	F	81	N/A	
PD529	F	70	N/A	

Supplementary Table 1. *Details of the human brain tissue of PD, ILBD and control cases used in this study. Braak stage not applicable in control patients. For each patient, striatum and substantia nigra tissue was collected.*

		Glycan								
		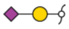		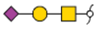		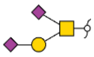		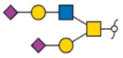		
		NeuNAcα2-3Gal **(Peeling structure)**		NeuNAcα2-3Galβ1-3GalNAc		NeuNAcα2-3Galβ1-3[NeuNAcα2-3]GalNAc		NeuNAcα2-3galβ1-3[NeuNAcα2-3Galβ1-4GlcNAcβ1-6]GalNAc		
**Stage**	**Method**	**% Area**	**CV**	**% Area**	**CV**	**% Area**	**CV**	**% Area**	**CV**	**Comments**
**OPTMIZED (Reduce/Alkylate, PNGase F digest, Crushed Gel, 12-minute, 70°C, 0.1 g/mL $(NH_4)_2CO_3$, pH 7.0 Neutralize, Desalt)**	**Fully Optimized**	**11.51**	**11.43**	**50.27**	**1.01**	**32.68**	**0.96**	**5.55**	**8.83**	**Fully optimized control**
OVEN-BASED RELEASE	Oven 16 Hours	85.98	0.72	10.06	1.44	3.35	10.15	0.62	20.97	High peeling
	Oven 40 Hours	87.51	1.07	10.49	6.44	1.40	15.00	0.61	8.20	
PRE-RELEASE	No Reduce/Alkylate	29.46	5.38	42.66	0.48	23.34	4.52	4.56	7.14	Inhibits *N*-release
	No PNGase F	14.49	6.14	44.93	2.00	33.23	0.11	7.36	23.86	Many surrounding peaks
	In Gel (Larger Gels)	11.21	2.77	46.81	4.28	35.00	6.00	6.99	3.08	Poorer absorption
	In Solution	30.34	1.75	44.26	0.07	21.97	1.91	3.43	4.08	Higher yield
RELEASE	5 Minute Reaction	12.45	2.65	43.81	1.81	36.87	4.81	6.88	19.13	Performed in separate experiments on a different day
	8 Minute Reaction	5.20	10.58	26.07	20.89	48.50	1.88	20.24	19.74	
	**12 Minute Reaction**	**4.46**	**6.50**	**30.35**	**2.41**	**47.61**	**0.05**	**17.58**	**2.39**	
	15 Minute Reaction	5.16	20.85	26.37	5.33	49.97	2.88	18.51	6.03	
	60°C Reaction	9.23	0.60	35.60	2.11	42.01	0.48	13.17	6.76	Performed in separate experiments on a different day
	**70°C Reaction**	**7.52**	**0.40**	**34.25**	**0.98**	**44.88**	**0.91**	**13.37**	**5.35**	
	80°C Reaction	10.83	6.46	31.50	2.57	41.07	1.78	16.60	5.06	
	0 g/mL $(NH_4)_2CO_3$	60.52	4.73	19.11	4.74	16.38	16.03	4.01	28.59	Increasing $(NH_4)_2CO_3$ produces growing peak at GU 1.9. Potential increase of core 1 structure
	0.05 g/mL $(NH_4)_2CO_3$	37.25	15.52	39.68	4.86	19.87	15.80	3.20	22.19	
	0.15 g/mL $(NH_4)_2CO_3$	12.17	9.25	48.11	1.13	33.63	1.49	6.09	19.21	
	0.2 g/mL $(NH_4)_2CO_3$	10.58	0.19	47.09	11.04	35.74	9.32	6.59	28.78	
POST-RELEASE	Neutralize pH 6.5	28.44	1.72	42.19	6.90	23.61	12.94	5.77	6.07	Degraded glycans?
	Neutralize pH 7.5	15.47	4.40	41.88	3.27	32.07	2.09	10.58	12.85	Peeling might still occur
	No Neutralize/Desalt	-	-	-	-	-	-	-	-	No discernable peaks
	Neutralize/No Desalt	84.74	3.88	9.11	22.02	4.91	20.16	1.25	23.20	High Peeling
	No Neutralize/No Desalt	85.41	0.40	8.29	5.13	5.18	12.36	1.13	10.22	

Supplementary Table 2. *% Areas of major fetuin O-glycan structures including the peeled glycan structure – NeuNAcα2-3Gal with coefficients of variation (CV; n=2) for each parameter tested. Optimal results highlighted in bold. Each experiment deviates from optimized protocol by the variable described in the method column. Symbol nomenclature is according to SNFG.*

Peak	Replicate 1	Replicate 2	Replicate 3	Replicate 4	Replicate 5	Replicate 6	Avg	Std Dev	CV
1	15.83	11.48	12.33	16.45	12.44	16.31	14.14	2.09	14.76
2	8.65	7.56	6.94	6.60	6.55	6.07	7.06	0.84	11.91
3	12.07	13.66	14.89	12.58	15.31	13.56	13.68	1.15	8.41
4	4.35	4.61	4.49	3.46	3.87	3.26	4.01	0.51	12.85
5	10.83	11.01	11.03	10.22	12.25	10.11	10.91	0.70	6.42
6	4.15	3.82	4.29	3.87	4.97	4.03	4.19	0.38	9.17
7	2.34	2.68	2.58	2.50	2.70	3.27	2.68	0.29	10.85
8	25.17	29.45	26.85	27.35	28.63	29.69	27.86	1.58	5.67
9	5.22	5.29	5.07	5.16	4.80	5.41	5.16	0.19	3.72
10	11.39	10.44	11.55	11.81	8.49	8.28	10.33	1.44	13.92

Supplementary Table 3. *Coefficient of Variation calculation for 10 major peaks in brain tissue non-reductive glycan release reproducibility test.*

Peak	GU	SNFG	Structure	Method of Confirmation
P1	0.98		GalNAc	Exo product
P2	1.02		Glc	Exo product
P3	1.04		Gal	Exo product
P4	1.70		SO3-Galβ1-3GalNAc	Exo + Ion Trap + QToF
	1.70		SO3-(NeuAcα2-6)GalNAc	Exo + QToF
P5	1.76		NeuAc-NeuAc	Exo + QToF
P6	1.78		Galβ1-3GalNAc	Exo + Ion Trap
	1.78		Galα1-3GalNAc	Exo + Ion Trap
	1.79		GlcNAcβ1-3GalNAc	Exo + Ion Trap + QToF
P7	2.42		NeuAcα2-6GalNAc	Exo + Ion Trap + QToF
P8	2.42		NeuAcα2-3Gal	Exo + QToF
P9	2.56		Galβ1-3GlcNAcβ1-2Man	Exo + Ion Trap + QToF
P10	2.62		Galβ1-4GlcNAcβ1-2Man	Exo + Ion Trap + QToF
P11	2.64		Galβ1-3[GlcNAcβ1-6]GalNAc	Exo
P12	2.71		GlcNAcβ1-3Galβ1-3GalNAc	Exo
P13	2.77		GlcNAcβ1-4Galβ1-3GalNAc	Exo
P14	2.81		Galβ1-3GlcNAcβ1-3GalNAc	Exo
P15	2.96		NeuAcα2-6Galβ1-3HexNAc	Exo + QToF
P16	3.05		NeuAcα2-3Galβ1-3GalNAc	Exo + Ion Trap + QToF
P17	3.23		SO3-Galβ1-4(Fucα1-3GlcNAcβ1-2)Man	Exo + Ion Trap + QToF
	3.27		(Fucα1-2Galβ1-3)(Fucα1-4HexNAc)	Exo
P18	3.28		Galβ1-4(Fucα1-3GlcNAcβ1-2)Man	Exo + Ion Trap + QToF
	3.28		Galβ1-3[Galβ1-4GlcNAcβ1-6]GalNAc	Exo
P19	3.38		Galβ1-3(NeuAcα2-6)GalNAc	Exo + Ion Trap + QToF
P20	3.40		SO3-Galβ1-3[GlcNAcβ1-6]GalNAc	Exo + QToF
P21	3.45		Galβ1-4(SO3-Fucα1-3GlcNAcβ1-2)Man	Exo + Ion Trap + QToF
P22	3.50		SO3-Galβ1-3[NeuAcα2-6]GalNAc	Exo + Ion Trap
P23	3.52		Galβ1-4GlcNAcβ1-3Galβ1-3GalNAc	Exo
	3.56		Galβ1-3GlcNAcβ1-3Galβ1-3GalNAc	Exo
P24	3.63		Fucα1-3-GlcNAcβ1-2[GlcNAcβ1-6]Man	Exo
P25	3.63		NeuAcα2-6Galβ1-3GlcNAcβ1-2Man	Exo + Ion Trap + QToF
	3.68		SO3-Galβ1-3[SO3-Galβ1-4GlcNAcβ1-6]GalNAc	Exo + QToF
	3.69		Galβ1-3[(Fucα1-3GlcNAcβ1-6)]GalNAc	Exo
	3.72		NeuAcα2-3Galβ1-4GlcNAcβ1-2Man	Exo + Ion Trap + QToF
	3.74		NeuAcα2-6Galβ1-4GlcNAcβ1-2Man	Exo + Ion Trap + QToF
P26	3.78		NeuAcα2-3Galβ1-3GlcNAcβ1-2Man	Exo + Ion Trap + QToF
P27	3.95		Galβ1-3[SO3-NeuAcα2-6]GalNAc	Exo + Ion Trap
P28	4.03		Galβ1-3[GlcNAcβ1-XGalβ1-4GlcNAcβ1-6]GalNAc	Exo
	4.05		GalNAcβ1-4GlcNAcβ1-2[GalNAcβ1-4GlcNAcβ1-6]Man	Exo + QToF
P29	4.05		Galβ1-3GlcNAcβ1-4Galβ1-3[GlcNAcβ1-6]GalNAc	Exo
	4.09		(Fucα1-3GlcNAcβ1-2)[(Fucα1-3GlcNAcβ1-6)]Man	Exo
P30	4.13		Galβ1-4(Fucα1-3-GlcNAcβ1-2)[GlcNAcβ1-6]Man	Exo + QToF
	4.13		GlcNAcβ1-3Galβ1-3[Galβ1-4GlcNAcβ1-6]GalNAc	Exo
P31	4.15		Galβ1-3[GlcNAcβ1-XGalβ1-4GlcNAcβ1-6]GalNAc	Exo

Supplementary Table 4. *O-glycans characterized across all PD and control groups. Glycans highlighted in green are the major component of each HILIC peak. Method of characterization confirmation is shown as such – Exo - confirmed with exoglycosidase panel, Ion Trap - confirmed mass and generic structure with PGC-LC-MS^n, QToF - confirmed mass with FLR-HILIC-UPLC-MS. Symbol nomenclature is according to SNFG.*

Peak	GU	SNFG	Structure	Method of Confirmation
P32	4.40	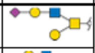	Galβ1-3[NeuAcα2-3Galβ1-4GlcNAcβ1-6]GalNAc	Exo + Ion Trap + QToF
	4.40	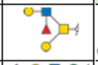	Galβ1-3[Galβ1-4(Fucα1-3GlcNAcβ1-6)]GalNAc	Exo + Ion Trap + QToF
P33	4.42	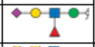	NeuAcα2-3Galβ1-4(Fucα1-3GlcNAcβ1-2)Man	Exo + QToF
	4.46	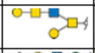	Galβ1-3[Galβ1-3GalNAcβ1-4GlcNAcβ1-6]GalNAc	Exo
	4.48	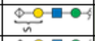	SO3-HexAβ1-3Galβ1-4GlcNAcβ1-2Man	Exo + Ion Trap + QToF
P34	4.54	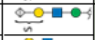	SO3-HexAβ1-3Galβ1-3GlcNAcβ1-2Man	Exo + Ion Trap + QToF
P35	4.57	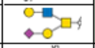	NeuAcα2-3Galβ1-3[Galβ1-4GlcNAcβ1-6]GalNAc	Exo + Ion Trap
	4.61	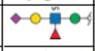	NeuAcα2-6Galβ1-4(Fucα1-3-SO3-GlcNAcβ1-2)Man	Exo
P36	4.70	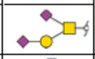	NeuAcα2-3Galβ1-3(NeuAcα2-6)GalNAc	Exo + Ion Trap + QToF
	4.75	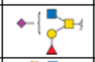	NeuAcα2-6-(Fucα1-2Galβ1-3)[GlcNAcβ1-6]GalNAc	Exo
P37	4.85	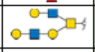	Galβ1-4GlcNAcβ1-4Galβ1-3[Galβ1-4GlcNAcβ1-6]GalNAc	Exo + QToF
P38	4.89	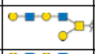	Galβ1-3[Galβ1-4GlcNAcβ1-4Galβ1-4GlcNAcβ1-6]GalNAc	Exo + QToF
	4.92	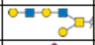	Galβ1-3[Galβ1-3GlcNAcβ1-4Galβ1-4GlcNAcβ1-6]GalNAc	Exo + QToF
P39	5.01	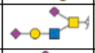	NeuAcα2-3Galβ1-3GlcNAcβ1-3[NeuAcα2-6]GalNAc	Exo + QToF
P40	5.13	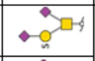	NeuAcα2-3(SO3-)Galβ1-3(NeuAcα2-6)GalNAc	Exo + Ion Trap + QToF
P41	5.13	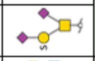	NeuAcα2-3(SO3-)Galβ1-3(NeuAcα2-6)GalNAc	Exo + Ion Trap + QToF
P42	5.23	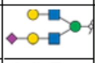	NeuAcα2-3Galβ1-4GlcNAcβ1-2[Galβ1-4GlcNAcβ1-6]Man	Exo
P43	5.24	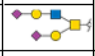	NeuAcα2-3Galβ1-3[NeuAcα2-6Galβ1-4GlcNAcβ1-6]GalNAc	Exo + Ion Trap
P44	5.30	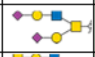	NeuAcα2-3Galβ1-3[NeuAcα2-3Galβ1-4GlcNAcβ1-6]GalNAc	Exo + Ion Trap
P45	5.57	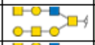	Galβ1-3GalNAcβ1-3Galβ1-3[GalNAcβ1-3Galβ1-4GlcNAcβ1-6]GalNAc	Exo
	5.61	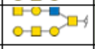	Galβ1-4GalNAcβ1-3Galβ1-3[GalNAcβ1-3Galβ1-4GlcNAcβ1-6]GalNAc	Exo
P46	5.65	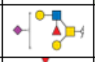	NeuAcα2-6-Galβ1-3[Galβ1-4(Fucα1-3GlcNAcβ1-6)]GalNAc	Exo + QToF
	5.68	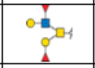	Fucα1-2Galβ1-3[Galβ1-4(Fucα1-3GlcNAcβ1-6)]GalNAc	Exo + QToF
	5.79	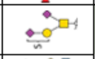	[SO3] + NeuAcα2-3Galβ1-3(NeuAcα2-6)GalNAc	Exo + Ion Trap + QToF
P47	5.83	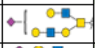	NeuAcα2-3-Galβ1-3GlcNAcβ1-4Galβ1-3[Galβ1-4GlcNAcβ1-6]GalNAc	Exo
	5.87	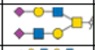	NeuAcα2-3GlcNAcβ1-4Galβ1-3[NeuAcα2-3Galβ1-4GlcNAcβ1-6]GalNAc	Exo
P48	5.89	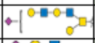	NeuAcα2-3-Galβ1-3[Galβ1-3GlcNAcβ1-4Galβ1-4GlcNAcβ1-6]GalNAc	Exo
	5.92	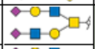	NeuAcα2-6GlcNAcβ1-4Galβ1-3[NeuAcα2-3Galβ1-4GlcNAcβ1-6]GalNAc	Exo
P49	5.94	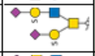	NeuAcα2-3-SO3-Galβ1-3[NeuAcα2-3-SO3-Galβ1-4GlcNAcβ1-6]GalNAc	Exo
P50	6.20	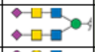	NeuAcα2-3GalNAcβ1-4GlcNAcβ1-2[NeuAcα2-3GalNAcβ1-4GlcNAcβ1-6]M	Exo + QToF
P51	6.41	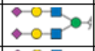	NeuAcα2-3Galβ1-4GlcNAcβ1-2[NeuAcα2-3Galβ1-4GlcNAcβ1-6]Man	Exo + Ion Trap
	6.47	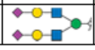	NeuAcα2-3Galβ1-4GlcNAcβ1-2[NeuAcα2-6Galβ1-4GlcNAcβ1-6]Man	Exo + Ion Trap

Supplementary Table 4 (cont.). *O-glycans characterized across all PD and control groups. Glycans highlighted in green are the major component of each HILIC peak. Method of characterization confirmation is shown as such – Exo - confirmed with exoglycosidase panel, Ion Trap - confirmed mass and generic structure with PGC-LC-MS^n, QToF - confirmed mass with FLR-HILIC-UPLC-MS. Symbol nomenclature is according to SNFG.*

Supplementary Table 5. *Exoglycosidase digestion panel for healthy striatum pool. – REFER TO SUPPLEMENTARY SPREADSHEET FILE.*

Supplementary Table 6. *Exoglycosidase digestion panel for PD (stages 1-4) striatum pool. – REFER TO SUPPLEMENTARY SPREADSHEET FILE.*

Supplementary Table 7. *Exoglycosidase digestion panel for healthy substantia nigra pool. – REFER TO SUPPLEMENTARY SPREADSHEET FILE.*

Supplementary Table 8. *Exoglycosidase digestion panel for PD (stages 1-4) substantia nigra pool. – REFER TO SUPPLEMENTARY SPREADSHEET FILE.*

Supplementary Table 9. *PGC-LC-MS^n data outlining m/z values as well as relative intensities and charge state observed. FLR-HILIC-UPLC-MS data outlining m/z values as well as relative charge state observed, error (ppm) and RT (TIC) of glycans. FLR-HILIC-UPLC data of 2AB labelled glycans outlining GU values and relative area. "x" – Gal core contamination. "nd" – not detected. – REFER TO SUPPLEMENTARY SPREADSHEET FILE.*

Component	**Short-Hand Notation**
Galactose (Gal) (β1-3/4/6 linked)	ga
Glucose (Glc) (β1-3/4/6 linked)	gl
N-acetylgalactosamine (GalNAc) (β1-3/4/6 linked)	GA
N-acetylglucosamine (GlcNAc) (β1-3/4/6 linked)	GL
Mannose (Man)	m
Fucose (Fuc) (α1-2/3 linked)	fa
Sulfate; SO3 (written directly before the monosaccharide)	s
Sialic acid (α2-3/6 linked)	S
Glucuronic acid (GlcA) (β1-3/4/6 linked)	U
Bold lettering	Core monosaccharide of the glycan
Inside square brackets	Right chain from core
Outside square brackets	Left chain from core
No square brackets	Linear glycan with no branching
Inside round brackets	Rightmost monosaccharide is in the chain, leftmost monosaccharide branches from it
α1-2 linked	2
α1/2-3, β1-3 linked	3
β1-4 linked	4
α1/2-6, β1-6 linked	6

Supplementary Table 10. *Short-hand naming convention of O-glycans used in the supplementary Tables 5-9.*

**Feature**	**Under 85**				**85+**			
	**Healthy vs PD Striatum**	**Healthy vs PD Nigra**	**Healthy Striatum vs Nigra**	**PD Striatum vs Nigra**	**Healthy vs PD Striatum**	**Healthy vs PD Nigra**	**Healthy Striatum vs Nigra**	**PD Striatum vs Nigra**
**Sialylation**	0.157	0.406	0.572	0.328	0.008	0.395	0.964	0.281
**Fucosylation**	0.909	1.000	0.813	1.000	0.999	0.837	0.276	0.954
**Sulfation**	0.202	1.000	1.000	0.646	0.050	0.992	0.997	0.733
**Mannose Core**	0.953	1.000	0.964	1.000	1.000	1.000	0.385	0.876
**Glucuronylation**	0.999	0.924	0.442	0.887	0.996	0.998	0.155	0.410
**Peeling**	0.086	0.772	1.000	0.925	0.111	0.973	1.000	0.759

Supplementary Table 11. *P-values derived from a comparison of glycan features across different groups comparing disease states separated by age ("under 85" and "85+"). Red indicates a decrease in relative abundance in the leading group (e.g the healthy group in healthy vs. PD).*

Feature	Healthy vs Diseased Striatum		ILBD vs PD Striatum	Healthy vs Diseased Nigra		ILBD vs PD Substantia Nigra	Healthy Striatum vs Nigra	Diseased Striatum vs Nigra	
	ILBD	PD		ILBD	PD			ILBD	PD
Sialylation	0.316	0.117	0.002	0.523	0.987	0.972	0.988	0.083	0.331
Fucosylation	0.369	1.000	0.632	1.000	1.000	1.000	0.855	0.999	0.999
Sulfation	1.000	0.022	0.101	1.000	1.000	1.000	0.933	1.000	0.417
Mannose Core	0.083	1.000	0.224	1.000	1.000	1.000	0.742	0.908	0.996
Glucuronylation	0.196	0.997	0.081	1.000	0.999	1.000	0.280	0.996	0.674
Peeling	0.898	0.137	0.036	0.785	0.994	0.996	1.000	0.161	0.945

Supplementary Table 12. *P-values derived from comparison of glycan features across different groups comparing disease states from females. Red indicates a decrease in relative abundance in the leading group (e.g the healthy group in healthy vs. PD) and green indicates an increase – only statistically significant areas highlighted. Main glycan of each peak used for analysis.*

Feature	Healthy vs Diseased Striatum		ILBD vs PD Striatum	Healthy vs Diseased Nigra		ILBD vs PD Substantia Nigra	Healthy Striatum vs Nigra	Diseased Striatum vs Nigra	
	ILBD	PD		ILBD	PD			ILBD	PD
Sialylation	-	0.060	-	-	0.030	-	0.465	-	0.507
Fucosylation	-	1.000	-	-	0.987	-	0.545	-	1.000
Sulfation	-	0.305	-	-	0.675	-	1.000	-	0.943
Mannose Core	-	0.999	-	-	1.000	-	0.990	-	1.000
Glucuronylation	-	1.000	-	-	0.979	-	0.400	-	0.887
Peeling	-	0.260	-	-	0.734	-	1.000	-	0.930

Supplementary Table 13. *P-values derived from comparison of glycan features across different groups comparing disease states from males. Red indicates a decrease in relative abundance in the leading group (e.g the healthy group in healthy vs. PD) and green indicates an increase – only statistically significant areas highlighted. Main glycan of each peak used for analysis. Cells marked with a "-" have no data due to the absence of males in ILBD group.*

References

(1) Furuki, K.; Toyo'oka, T.; Ban, K., Highly sensitive glycosylamine labelling of O-glycans using non-reductive β-elimination. *Anal. Bioanal. Chem.* **2017,** *409* (9), 2269-2283.

(2) Kozak, R. P.; Tortosa, C. B.; Fernandes, D. L.; Spencer, D. I., Comparison of procainamide and 2-aminobenzamide labeling for profiling and identification of glycans by liquid chromatography with fluorescence detection coupled to electrospray ionization-mass spectrometry. *Anal. Biochem.* **2015,** *486*, 38-40.

(3) Keser, T.; Pavić, T.; Lauc, G.; Gornik, O., Comparison of 2-aminobenzamide, Procainamide and RapiFluor-MS as derivatizing agents for high-throughput HILIC-UPLC-FLR-MS N-glycan analysis. *Front. Chem.* **2018,** *6*, 324.

(4) Royle, L.; Mattu, T. S.; Hart, E.; Langridge, J. I.; Merry, A. H.; Murphy, N.; Harvey, D. J.; Dwek, R. A.; Rudd, P. M., An analytical and structural database provides a strategy for sequencing O-glycans from microgram quantities of glycoproteins. *Anal. Biochem.* **2002,** *304* (1), 70-90.

(5) Guile, G. R.; Harvey, D. J.; O'Donnell, N.; Powell, A. K.; Hunter, A. P.; Zamze, S.; Fernandes, D. L.; Dwek, R. A.; Wing, D. R., Identification of highly fucosylated N-linked oligosaccharides from the human parotid gland. *Eur. J. Biochem.* **1998,** *258* (2), 623-656.

(6) Huang, Y.; Mechref, Y.; Novotny, M. V., Microscale nonreductive release of O-linked glycans for subsequent analysis through MALDI mass spectrometry and capillary electrophoresis. *Anal. Chem.* **2001,** *73* (24), 6063-6069.

(7) Kozak, R. P.; Royle, L.; Gardner, R. A.; Bondt, A.; Fernandes, D. L.; Wuhrer, M., Improved nonreductive O-glycan release by hydrazinolysis with ethylenediaminetetraacetic acid addition. *Anal. Biochem.* **2014,** *453*, 29-37.

(8) Yang, S.; Höti, N.; Yang, W.; Liu, Y.; Chen, L.; Li, S.; Zhang, H., Simultaneous analyses of N-linked and O-linked glycans of ovarian cancer cells using solid-phase chemoenzymatic method. *Clin. Proteomics* **2017,** *14*, 3-3.

(9) Karlsson, N. G.; Packer, N. H., Analysis of O-linked reducing oligosaccharides released by an in-line flow system. *Anal. Biochem.* **2002,** *305 2*, 173-85.

(10) Ye, Z.; Mao, Y.; Clausen, H.; Vakhrushev, S. Y., Glyco-DIA: a method for quantitative O-glycoproteomics with in silico-boosted glycopeptide libraries. *Nat. Methods* **2019,** *16* (9), 902-910.
